# Influence of ethnic traditional cultures on genetic diversity of rice landraces under on-farm conservation in southwest China

**DOI:** 10.1186/s13002-016-0120-0

**Published:** 2016-10-27

**Authors:** Yanjie Wang, Yanli Wang, Xiaodong Sun, Zhuoma Caiji, Jingbiao Yang, Di Cui, Guilan Cao, Xiaoding Ma, Bing Han, Dayuan Xue, Longzhi Han

**Affiliations:** 1 Institute of Crop Science, Chinese Academy of Agriculture Sciences, Beijing, 100081 China; 2 College of Life and Environmental Sciences, Minzu University of China, Beijing, 100081 China; 3 Inner Mongolia Institute of Biotechnology Research, Hohhot, 010070 China; 4 Heilongjiang Institute of Sericulture Research, Harbin, 150086 China

**Keywords:** Ethnic traditional cultures, *Ex-situ* conservation, Genetic diversity, On-farm conservation, Rice landraces

## Abstract

**Background:**

Crop genetic resources are important components of biodiversity. However, with the large-scale promotion of mono-cropping, genetic diversity has largely been lost. *Ex-situ* conservation approaches were widely used to protect traditional crop varieties worldwide. However, this method fails to maintain the dynamic evolutionary processes of crop genetic resources in their original habitats, leading to genetic diversity reduction and even loss of the capacity of resistance to new diseases and pests. Therefore, on-farm conservation has been considered a crucial complement to *ex-situ* conservation. This study aimed at clarifying the genetic diversity differences between *ex-situ* conservation and on-farm conservation and to exploring the influence of traditional cultures on genetic diversity of rice landraces under on-farm conservation.

**Methods:**

The conservation status of rice landrace varieties, including *Indica* and *Japonica*, non-glutinous rice (*Oryza sativa*) and glutinous rice (*Oryza sativa* var. glutinosa Matsum), was obtained through ethno-biology investigation method in 12 villages of ethnic groups from Guizhou, Yunnan and Guangxi provinces of China. The genetic diversity between 24 pairs of the same rice landraces from different times were compared using simple sequence repeat (SSR) molecular markers technology. The landrace paris studied were collected in 1980 and maintained *ex-situ*, while 2014 samples were collected on-farm in southwest of China.

**Results:**

The results showed that many varieties of rice landraces have been preserved on-farm by local farmers for hundreds or thousands of years. The number of alleles (Na), effective number of alleles (Ne), Nei genetic diversity index (He) and Shannon information index (I) of rice landraces were significantly higher by 12.3–30.4 % under on-farm conservation than under *ex-situ* conservation. Compared with the *ex-situ* conservation approach, rice landraces under on-farm conservation programs had more alleles and higher genetic diversity. In every site we investigated, ethnic traditional cultures play a positive influence on rice landrace variety diversity and genetic diversity.

**Conclusion:**

Most China’s rice landraces were conserved in the ethnic areas of southwest China. On-farm conservation can effectively promote the allelic variation and increase the genetic diversity of rice landraces over the past 35 years. Moreover, ethnic traditional culture practices are a crucial foundation to increase genetic diversity of rice landraces and implement on-farm conservation.

**Electronic supplementary material:**

The online version of this article (doi:10.1186/s13002-016-0120-0) contains supplementary material, which is available to authorized users.

## Background

Crop genetic resources are important components of biodiversity. These resources play a crucial role in the Chinese economy and food security, since more than 50 % of the population depends on agriculture for their livelihood. However, with the advent of biotechnology and the large-scale promotion of mono-cropping, genetic diversity has largely been lost [1]. It is estimated that worldwide agriculture has lost 75 % of the genetic diversity of major food crops between 1900 and 2000, a process that is continuing at an annual rate of 1–2 % [2]. To protect these valuable genetic resources, *ex-situ* conservation approaches such as cryopreservation, field gene banks, in vitro conservation, botanical gardens have been undertaken, which are widely used to protect traditional crop varieties worldwide [3, 4]. China National Gene Bank for long-term seed preservation was also built in Beijing in 1986 [5]. Approximately 0.4 million of crop germplasm resources were conserved in the National Gene Bank by the end of 2010, including more than 80,000 rice germplasm resources, two-thirds are rice landraces [6]. However, Gene Bank method fails to maintain the dynamic evolutionary processes of crop genetic resources in their original farm habitats, farmers lose the opportunity to select and manage crop varieties. In addition, genetic drift and gene mutations occur during the process of updating germplasm, eventually leading to genetic diversity reduction and even loss of the capacity of plants to adapt to new ecological environments and to develop resistance to new diseases and pests [7–9]. Therefore, an *in-situ* conservation strategy involving the participation of farmers, also known as on-farm conservation, has emerged and increasingly became a focus of study, as this strategy is considered to represent a crucial complement to *ex-situ* conservation [10–12].

Bellon et al. defined on-farm conservation of crop genetic resources as “the continued cultivation and management of a diverse set of crop populations by farmers in the agro-ecosystems where a crop has evolved” [7]. This conservation strategy not only maintains the natural mutation and diversity evolution of crop resources with the changing environment, but it also includes human selection and management, as well as the active roles of ethnic customs and traditional cultures in preserving crop landraces and increasing genetic diversity [13, 14]. This definition emphasizes the role of farmers in ultimately determining whether crop populations are maintained or abandoned. Since the 1990s, numerous studies have focused on the approach of on-farm protection, including the mechanism and theory of on-farm conservation [7, 15, 16], as well as case studies examining the influence of genetic diversity on different crop varieties, such as rice (*Oryza sativa*) [17], maize (*Zea mays*) [18], potato (*Solanum tuberosum*) [11], sorghum (*Sorghum bicolor*) [19], bean (*Phaseolus vulgaris*) [20], cassava (*Manihot esculenta*) [21], and other staple crops. Moreover, studies of the effectiveness of on-farm conservation projects involving native crops have been carried out all over the world, and found that considerable traditional crop genetic diversity continues to be maintained by a large number of small farms [22], some projects even generating positive outcomes [23].

There is a very close relationship between ethnic rice-cultivating cultures and rice variety diversity throughout the world. Understanding their cultural background is important for conserving the diversity of crop varieties [11, 24–27]. Asian farmers have been cultivating thousands of rice landraces with different aromas, tastes, medicinal properties and cultural values to meet their culinary and cultural requirements [28]. Mexican farmers in the Oaxaca region plant 11 different corn landraces used to produce 9 different types of dishes [29]. Traditional crop varieties in Ethiopia including African millet (*Eleusine coracana*), wheat (*Triticum turgidum*) [30] and barley [31] are well preserved to meet the needs of traditional local food cultures.

Compared to new rice cultivars, rice landraces have more complex genetic backgrounds and more abundant genetic diversity and heterogeneity, as well as strong adaptability to the environment, excellent resistance to diseases and pests, high yields and good quality [32]. The southwest of China, Guizhou, Yuannan and Guangxi provinces, is one of the largest center of rice genetic diversity and high quality germplasm in the world [33–35]. Microsatellite or simple sequence repeat (SSR) markers have emerged as powerful tools for analyzing genetic diversity and structure in rice, due to their independence from environmental conditions, stable expression and reproducibility. These markers currently are comparatively suitable indicators of gene variation between populations [36]. Related researchers analyzed the genetic diversity of different rice landraces in China using SSR molecular markers, successfully revealing changes in genetic diversity [37–39]. Most studies examining the influence of two conservation methods, i.e., on-farm and *ex-situ* conservation, on the genetic diversity of crop variety resources have focused on rice of different origins and varieties at the population level [40–45], However, only Sun et al. [39] and a few other researchers have performed comparative genetic analysis of the structures within single-origin pairs of crop landraces from on-farm and *ex-situ* conservation programs. Most of these studies have focused on Yunnan, while Guizhou and Guangxi have rarely been examined.

In this paper, we investigated 12 villages regarding the preservation status of rice landrace and traditional cultures impacts on rice landraces in southwest China, and selected 24 pairs of the same rice landraces under on-farm and *ex-situ* conservation to compare the genetic diversity using SSR molecular markers technology. The purpose of this study was (1) to evaluate the conservation status of rice landraces in ethnic group areas in southwest of China, (2) to clarify the differences of genetic diversity of rice landraces between *ex-situ* conservation and on-farm conservation over the past 35 years, (3) to explore the impact of traditional cultures on higher genetic diversity of rice landraces under on-farm conservation, and (4) to provide a guide for effective conservation of genetic diversity of rice landraces in these centers of cultivated rice diversity.

## Methods

### Field surveys

**Fig. 1:**
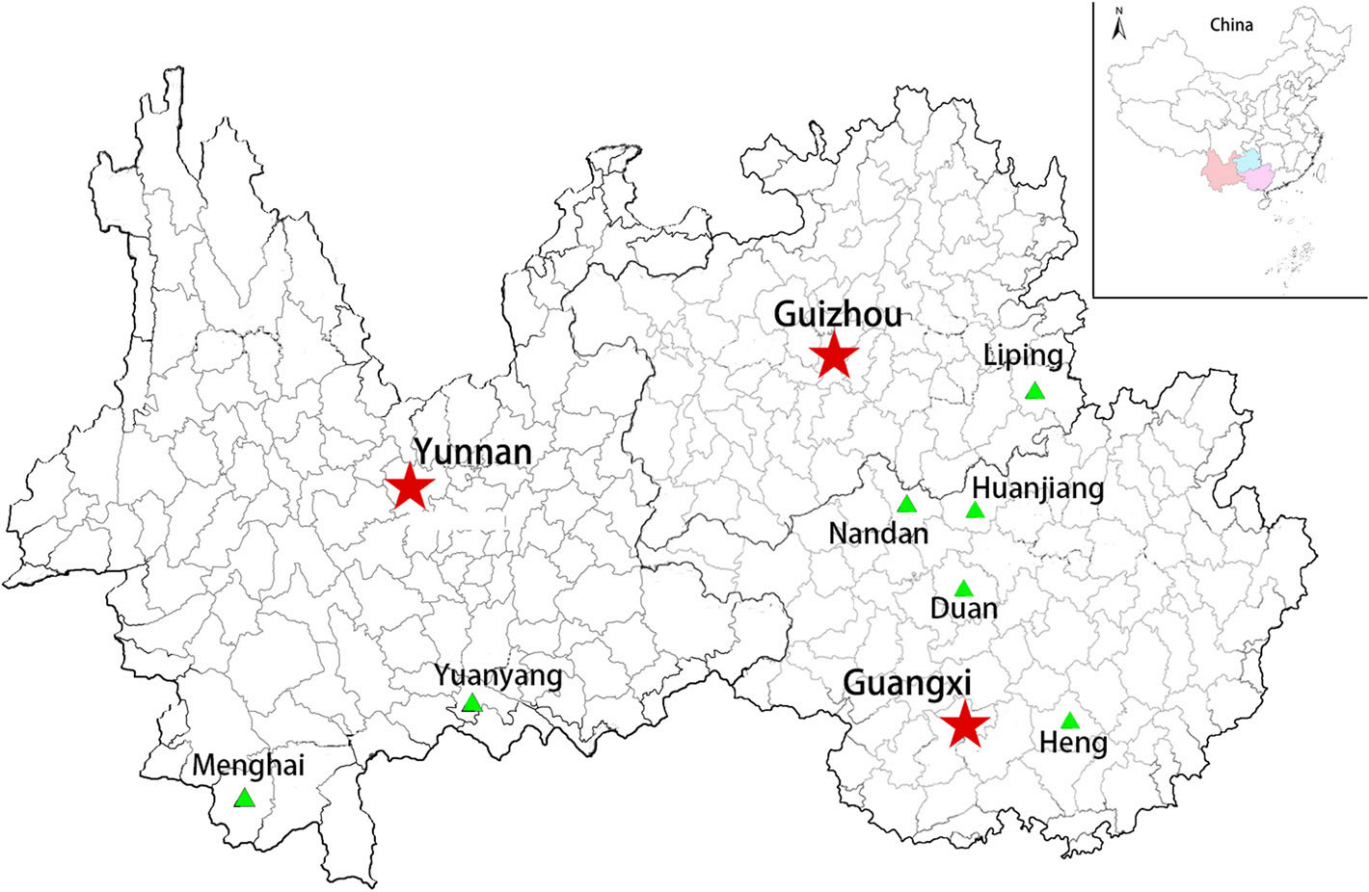
Distribution of rice landraces selected in Yunnan, Guizhou and Guangxi provinces of China

Rice landrace conservation status of ethnic groups was obtained through the methods of ethno-biological research. From October to November 2013 and June to July 2014, fieldwork collection was conducted in 12 villages distributed in 7 counties of Guizhou, Yunnan and Guangxi provinces (Fig. 1). The selected villages covering the altitude gradient, range of agro-ecological conditions, different minority ethnic group, the amount of conserved traditional rice landrace varieties. Therefore, the villages were selected to contain 9 minority ethnic groups, including Dong, Miao, Dai, Bulang, Lahu, Hani,Yao, Zhuang and Maonan people, and lots of rice landrace varieties including *Indica* and *Japonica*, non-glutinous rice (*Oryza sativa*) and glutinous rice (*Oryza sativa* var. glutinosa Matsum) had been cultivated in different altitude and climatic environment for hundreds of years. Rice landrace related variety quantity, utilization way and culture custom information were collected through different interview methods: participatory observation, semi-structured interviews, key informant interviews, focus group discussions and cultural anthropology [46, 47].

### Sampling methods

Rice landraces conserved *ex-situ* were obtained from the National Gene Bank, which were first collected in 1980. Rice landraces conserved on-farm were obtained from Guizhou, Yunnan and Guangxi provinces in 2014. Finally, we selected 48 varieties of rice landraces (24 pairs) sharing the same name and origin area that were collected at different times. The collection and sampling strategy of rice landraces in 1980 and in 2014 were similar with each other. For each variety, a bulk of seeds was collected through mixed sampling (more than 20 single plant), provided by a household. The varieties were collected either in home granaries or in farmers’ fields, depending on the harvesting time. For the 2014 rice population, we sampled all rice landrace varieties listed by a representative ethnic group of farmers in each village. Since the rice landraces collected in 1980 have maintained almost complete genetic integrity, the genetic differences between the same landraces were mainly due to on-farm conservation. The rice landraces under *ex-situ* conservation were updated only once, with updated population sizes of approximately 80 plants, which helped maintain the integrity of the populations as well [48]. In this study, seed samples collected in 1980 are called “germplasm name 1”, seed samples collected in 2014 are called “germplasm name 2”. The sizes of the rice landrace populations ranged from 44 to 52, with an average size of 48. The information of selected seed samples are shown in Table 1.

**Table 1:** Details of population size and origin region of rice landraces in different periods

Germplasm	Population size		Taxon	Locality (Village/Town/County/Province)
	2014	1980		
Baixianghe	44	52	*Japonica,* glutinous	Kengdong /Shuangjiang /Liping /Guizhou
Ronghe	45	47	*Japonica,* glutinous	Zaigong /Yandong /Liping /Guizhou
Danuo	46	46	*Japonica,* glutinous	Kengdong /Shuangjiang /Liping /Guizhou
Heinuo	45	48	*Japonica,* glutinous	Kengdong /Shuangjiang /Liping /Guizhou
Dalaogeng	48	48	*Indica,* non-glutinous	Quanfuzhuang /Xinjie /Yuanyang /Yunnan
Huangnuogu	48	47	*Indica,* non-glutinous	Huangcaoling /Xinjie /Yuanyang /Yunnan
Laogengbaijiao	48	48	*Indica,* non-glutinous	Qingkou /Xinjie /Yuanyang /Yunnan
Yaduogu	48	48	*Indica,* non-glutinous	Qingkou / Xinjie /Yuanyang /Yunnan
Laogenghongjiao	48	48	*Indica,* non-glutinous	Qingkou / Xinjie /Yuanyang /Yunnan
Huagu	46	48	*Indica,* non-glutinous	Adang zhai / Xinjie /Yuanyang /Yunnan
Lengshuigu	48	47	*Indica,* non-glutinous	Adang zhai / Xinjie /Yuanyang /Yunnan
Jiuyuenuo	48	48	*Japonica,* glutinous	Anfen zhai / Xinjie /Yuanyang /Yunnan
Xiangnuogu	48	48	*Indica,* non-glutinous	Liangxin /Niujiao zhai /Yuanyang /Yunnan
Nuogu1	48	48	*Indica,* non-glutinous	Danuoyou /Mengsong /Menghai /Yunnan
Nuogu2	48	48	*Indica,* non-glutinous	Danuoyou /Mengsong /Menghai /Yunnan
Xiaowuzui	44	47	*Indica,* non-glutinous	Danuoyou /Mengsong /Menghai /Yunnan
Dibaigu	48	47	*Indica,* non-glutinous	Hesong /Xiding /Menghai /Yunnan
Paozhugu	48	46	*indica,* non-glutinous	Mengwang /Basan /Menghai /Yunnan
Bendidanuo	48	47	*Japonica,* glutinous	Nonglv /Chengjiang /Duan /Guangxi
Honggu	48	48	*Indica,* non-glutinous	Chentang /Nan /Heng /Guangxi
Heigu	48	48	*Indica,* non-glutinous	Chentang /Nan /Heng /Guangxi
Xihongmi	48	48	*Indica,* non-glutinous	Sancha /Pingma /Heng /Guangxi
Danuo1	48	48	*Japonica,* glutinous	Xianan /Xianan /Huanjiang /Guangxi
Danuo2	48	48	*Japonica,* glutinous	Huaili /Lihu /Nandan /Guangxi

### SSR analysis

For each rice landrace variety collected, DNA was extracted from single leaf tissue at the tillering stage. 13 SSR markers distributed throughout the 11 rice chromosomes were preliminarily screened by consulting the results of Yang et al. [49], Sun et al. [39] and Cui et al. [50]. Details of the characterization of primers are available at www.gramene.org. The PCR amplifying procedure followed the procedure described by Panaud et al. [51], and subsequently run on a 6 % denatured polyacrylamide gel at 80 W. All of the gels were stained with the silver method as described by Bassam et al. [52].

### Genetic diversity data analysis

Genetic diversity was estimated by the number of alleles (Na), effective number of alleles (Ne), Nei’s genetic diversity index (He) and Shannon’s Information index (I) using POPGENE 32 [53, 54]. The significance of differences of Na, Ne, He and I between the same name populations collected in different periods was calculated using SPSS software. Populations from on-farm and *ex-situ* conservation were clustered using Unweighted Pair Group Method with Arithmetic (UPGMA) cluster system of NTSYS-pc V2.1. The genetic structure differentiation between populations of on-farm and *ex-situ* conservation programs was analyzed with the method of Analysis of Molecular Variance (AMOVA) implemented in the software package ARLEQUIN V 3.0 [55].

## Results

### Rice landraces conservation status in ethnic villages of Yunnan, Guizhou and Guangxi provinces

**Fig. 2:**
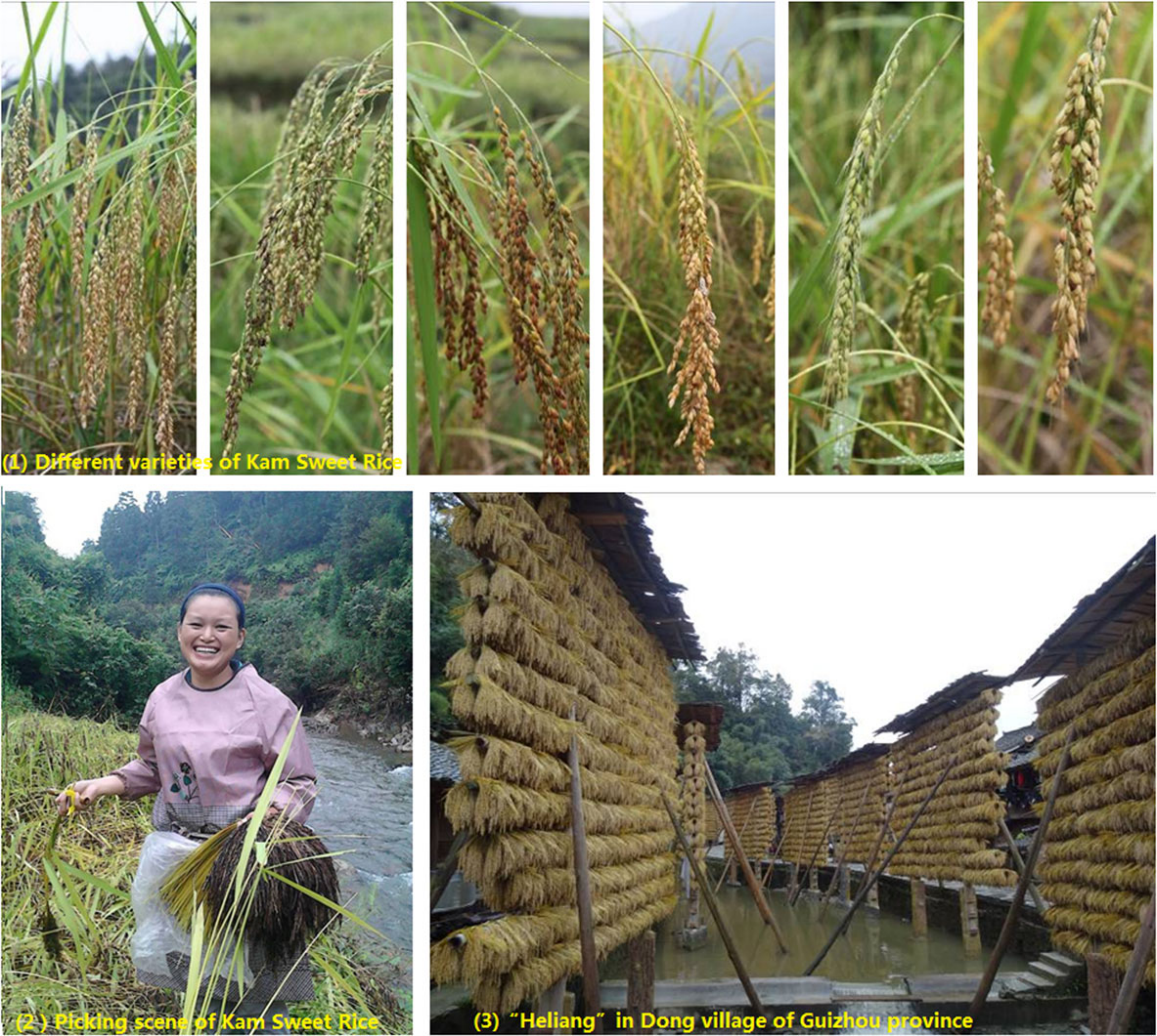
Different varieties of Kam Sweet Rice and harvesting scene by Dong people in Guizhou province

We investigated 12 minority villages in 7 counties from Guizhou, Yunnan and Guangxi provinces. The results showed that there were 60 varieties of rice landrace in total conserved in ethnic areas (Table 2). The local farmers have been continuing to conserve these rice landraces on farm for hundreds or thousands of years, and even some varieties are cultivated in large areas at present. Because the rice varieties were cultivated by different minority groups in different altitude (380–1811 m) and climatic environment, various specific characteristic of varieties and utilization method had been formed. In Liping county of Guizhou province, the “Kam Sweet Rice”, one glutinous rice variety, which has been cultivated and utilized by Dong and Miao people for more than 1,000 years. Kengdong and Huanggang, the investigated village, cultivated 19 varieties of Kam Sweet Rice. Different varieties of Kam Sweet Rice and harvesting scene are shown in Fig. 2. In Yuanyang Hani terrace of Yunnan province, 24 Red Rice varieties including Yuelianggu, Lengshuigu and so on, have been also conserved by Hani people in the high altitude areas for more than 1700 years. In Xishuangbanna tropical regions of Yunnan, 5 Dry Rice and glutinous varieties were cultivated in small areas by Dai, Bulang, Lahu and Hani people. In Guangxi province, 12 rice landraces conserved in 4 villages by Yao, Zhuang and Maonan people. Although a lot of rice landraces have been lost, many traditional rice varieties are still conserved by ethnic groups in southwest of China.

**Table 2:** Rice landraces conservation status in ethnic villages of Guizhou, Yunnan and Guangxi provinces

Village	Ethnic group	Altitude (m)	County /Province	Variety quantity	Rice landraces conserved
Kengdong	Dong	380	Liping /Guizhou	9	Wuminghe, Baixianghe, Heimanghe, Danuo, Niumaohe, Gonggenghe, Tonghe, Heinuo, Rongdonghe
Huanggang	Dong, Miao	735	Liping /Guizhou	10	Liezhuhe, Honghe, Jindongnuo, Yangnong, Bianlongtunuo, 60 days, 70 days, Shashupinuo, Baimangwanshunuo, Heimangwanshunuo
Qingkou	Hani	1600	Yuanyang /Yunnan	13	Yuelianggu, Huangnuogu, Hongnuogu, Wazhegu, Hangu, Chejie, Bozhugu, Yaduogu, Luxigu, Zigu, Chizugu, Baijiaolaogeng, Hongjiaolaogeng
Quanfuzhuang	Hani	1811	Yuanyang /Yunnan	6	Dalaogeng, Lvyegu, Chizugu, Jingu, Zhulugu, Huagu
Adang zhai	Hani	1730	Yuanyang /Yunnan	5	Lengshuigu, Mayigu,Honggu, Budaogu, Dalixiang
Huilaoxinzhai	Hani, Bulang	1165	Menghai/Yunnan	3	Nuogu, Manjingu, Hongmi,
Danuoyou	Dai, Lahu	817	Menghai/Yunnan	2	Xiaowuzui, Xiaomaogu
Nonglv	Zhuang	420	Duan/Guangxi	2	Bendidanuo, Nuogu
Huaili	Yao	583	Nandan/Guangxi	2	Zhainuo (Danuo), Danuo (Xiaonuo)
Chentang	Zhuang	420	Heng/Guangxi	2	Honggu, Heigu
Sancha	Zhuang	390	Heng/Guangxi	3	Xihongmi, Baikezi, Huangnuo
Xianan	Maonan	410	Huanjiang/Guangxi	3	Danuo, Heinuo, Gaogandanuo

**Fig. 3:**
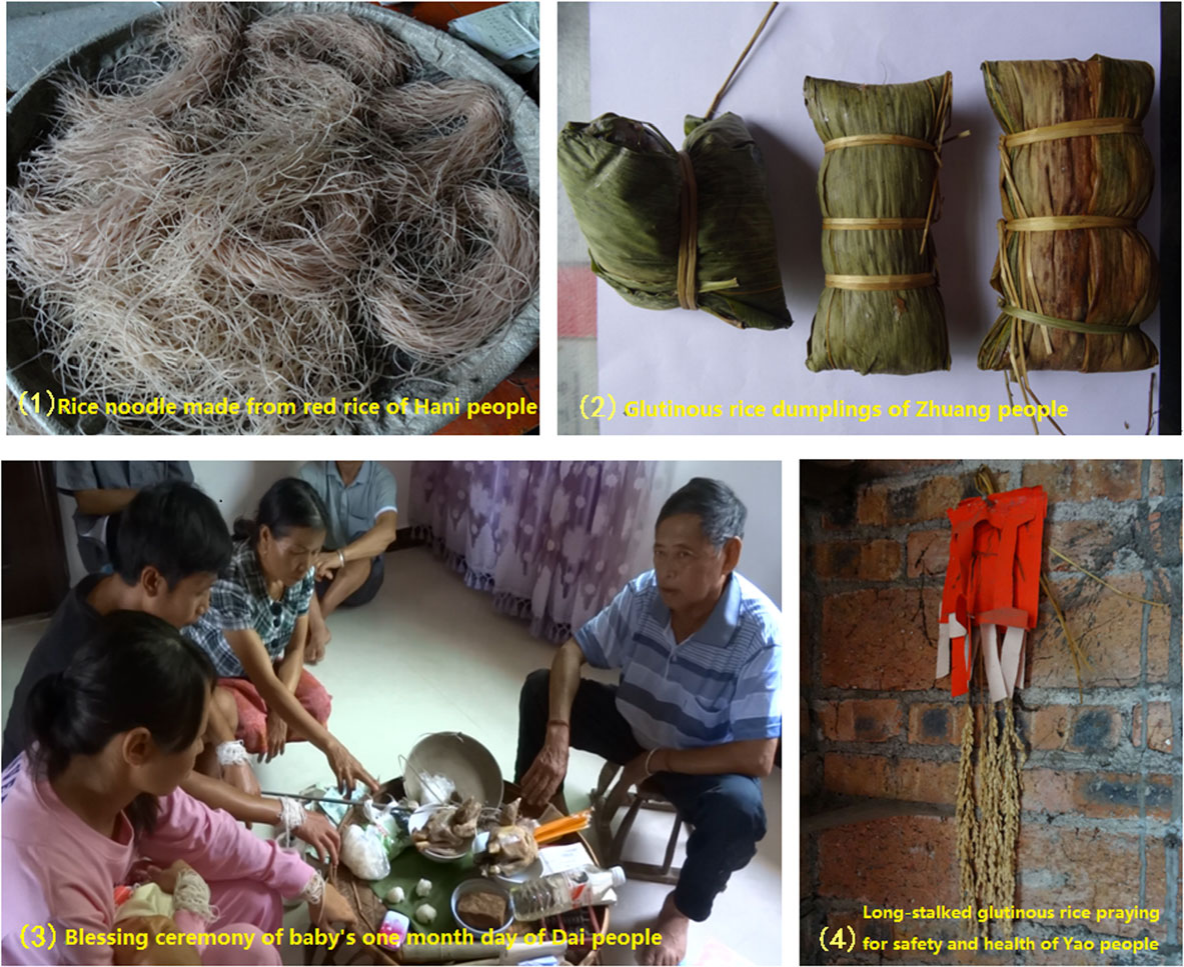
Rice landraces using way in ethnic traditional cultures and customs

People of different ethnic groups with their own traditional cultures have enriched the variety diversity of rice landrace. Rice landraces using way in some ethnic traditional cultures and customs are shown in Fig. 3. Dong people have rich variety resources of Kam Sweet Rice, not only the rice landraces are the necessary staple food, but also the indispensable for ethnic festival and religious ceremony. For instance, such as Hantian Day in Huanggang village, Dong people must make glutinous rice cake and glutinous rice wine with Liezhuhe or Honghe rice varieties used to sacrifice to the heaven. The Hani people in Yuanyang terrace promoted Red Rice as the essential materials in daily life. Moreover, in Hani's all kinds of ritual activities, such as worship to the ancestor, the forest god, the village god and many festivals including Spring Festival, Dragon Boat Festival, Torch Festival, weddings and funerals, traditional glutinous rice and red rice varieties are used to make zongzi, noodle, block bait and other traditional features food. Dai people are Buddhist ethnic group in Xishuangbanna, glutinous rice as the prerequisite tribute in temple activities and the cherish gift in baby’s one month day. It is precisely because traditional rice food are necessary in ethnic culture practices and customs, many rice landraces have been preserving for several decades, although 100–200 kg of rice are needed every year. Yao people in Guangxi province, put the ear of long-stalked rice on the wall or the door, it demonstrates people's wish of praying for safety and health for the family. Therefore, ethnic traditional culture practices and customs have a key role in conserving of traditional varieties and maintaining of crop genetic diversity on farm.

### Comparative genetic diversity within landrace pairs from on-farm and *ex-situ* conservation

We analyzed the genetic diversity and allelic polymorphisms within populations of the 24 rice landrace pairs under on-farm and *ex-situ* conservation using 13 pairs of microsatellite primers. The average Na, Ne, He and I values of rice landrace populations collected from farmlands and the Gene Bank are shown in Table 3. 17 rice landrace pairs were detected more alleles in the 2014 populations than the 1980 populations, accounting for 71 % of total samples (Including Xihongmi, the alleles of the 2014 populations were equal to the 1980 populations). The difference in allele count for Paozhugu between 2014 and 1980 was highly significant (*P* = 0.001). 6 paris of landraces (Laogenghongjiao, Lengshuigu, Jiuyuenuo, Xiaowuzui, Dibaigu, Bendidanuo), the number of alleles was significantly higher in the 2014 populations than in the 1980 populations. 13 pairs of landraces under on-farm conservation had higher Ne than those under *ex-situ* conservation, accounting for 54 % of the landraces. Within pairs of rice landraces, Lengshuigu, Paozhugu and Xiaowuzui from the 2014 populations had much higher Ne than the 1980 populations (*P* < 0.01), Ronghe, Laogenghongjiao and Danuo2 had significantly higher Ne than the 1980 populations (*P* < 0.05). 14 pairs of landraces (58 %) under on-farm conservation had higher He than those from Gene-Bank, with Lengshuigu and Paozhugu showing highly significant differences (*P* < 0.01), Ronghe, Laogenghongjiao, Xiaowuzui, Dibaigu and Danuo2 showed significant differences (*P* < 0.05). Half of the landraces from the 2014 population had higher Nei than the 1980 populations. A similar pattern was observed for I, with 14 landrace pairs under on-farm conservation having higher values than those collected in 1980, accounting for 58 % of landraces as well. The I values of Lengshuigu, Xiaowuzui and Paozhugu under on-farm conservation were much higher than those under *ex-situ* conservation (*P* < 0.01), while those of Ronghe, Laogenghongjiao, Dibaigu and Danuo2 were significantly difference between conservation practices (*P* < 0.05). These findings indicate that the genetic diversity of most rice landraces had increased under on-farm conservation.

**Table 3:** The average of Na, Ne, He and I comparisons of rice landraces between on-farm and *ex-situ* conservation

Landrace	Na		Ne		He		I	
	2014	1980	2014	1980	2014	1980	2014	1980
Baixianghe	2.92	1.85	1.532	1.392	0.264	0.203	0.51	0.332
Ronghe	2.23	1.85	1.347*	1.167*	0.186**	0.118**	0.333*	0.211*
Danuo	2.15	1.92	1.327	1.363	0.178	0.183	0.313	0.305
Heinuo	1.85	2.00	1.387	1.420	0.195	0.220	0.323	0.358
Dalaogeng	2.23	2.38	1.341	1.431	0.174	0.251	0.326	0.434
Huangnuogu	2.15	1.77	1.291	1.199	0.187	0.099	0.333	0.186
Laogengbaijiao	3.15	2.85	1.503	1.505	0.292	0.250	0.529	0.455
Yaduogu	3.00	2.85	1.846	1.680	0.307	0.311	0.582	0.551
Laogenghongjiao	2.92*	2.31*	1.535*	1.202*	0.285**	0.152**	0.522*	0.290*
Huagu	3.54	2.85	1.342	1.528	0.207	0.244	0.445	0.471
Lengshuigu	3.15*	1.38*	1.392**	1.096**	0.249**	0.082**	0.474**	0.167**
Jiuyuenuo	2.46*	1.62*	1.187	1.171	0.144	0.104	0.281	0.181
Xiangnuogu	2.38	2.08	1.378	1.317	0.185	0.183	0.342	0.332
Nuogu1	2.77	3.08	1.131	1.183	0.106	0.150	0.234	0.327
Nuogu2	1.38	1.69	1.092	1.131	0.051	0.086	0.088	0.153
Xiaowuzui	3.00*	2.00*	1.78**	1.27**	0.351*	0.127*	0.614**	0.228**
Dibaigu	3.00*	2.23*	1.523	1.229	0.235**	0.127**	0.458*	0.240*
Paozhugu	6.46**	2.31**	3.499**	1.372**	0.636**	0.240**	1.349**	0.407**
Bendidanuo	3.08*	1.77*	1.336	1.094	0.182	0.070	0.358	0.145
Honggu	2.15	2.38	1.274	1.439	0.156	0.242	0.271	0.430
Heigu	2.23	3.08	1.141	1.466	0.103	0.287	0.206	0.542
Xihongmi	1.85	1.85	1.127	1.244	0.084	0.147	0.155	0.248
Danuo1	1.85	2.00	1.187	1.275	0.136	0.178	0.247	0.319
Danuo2	1.92	1.46	1.435*	1.106*	0.246**	0.061**	0.392*	0.115*

*P < 0.01, significant difference **P < 0.05, obviously significant difference

### Evolution of alleles within rice landraces under on-farm and *ex-situ* conservation

**Fig. 4:**
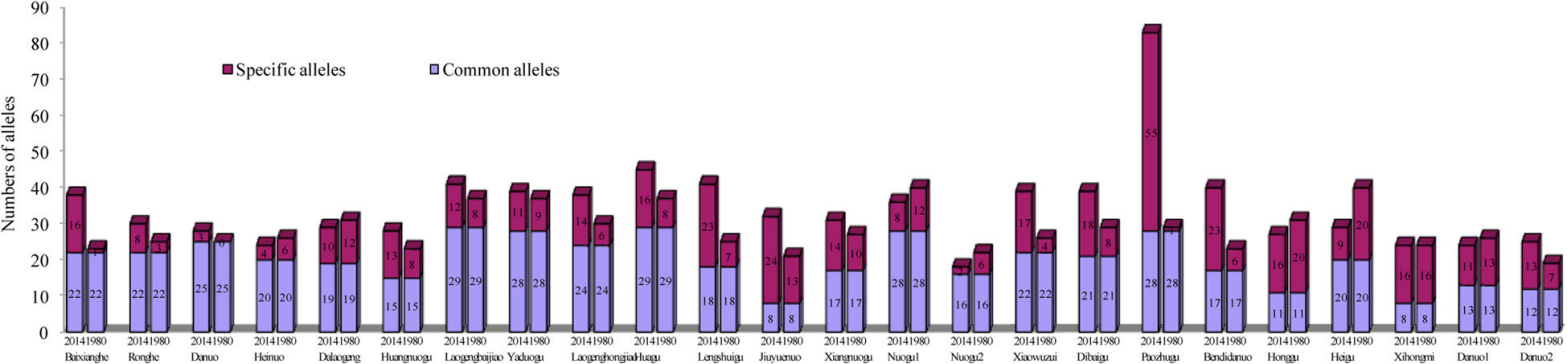
Change in numbers of alleles between the same name rice landraces populations collected in different periods

We compared the allelic composition of 24 rice landrace pairs under on-farm versus *ex-situ* conservation (Fig. 4). The number of common alleles detected in either the 1980 or 2014 populations ranged from 8 (Xihongmi and Jiuyuenuo) to 29 (Laogengbaijiao and Huagu), accounting for 25−64.6 % of total alleles detected in the 2014 populations and 33.3−78.4 % of total alleles detected in 1980 populations. Specific alleles of rice landraces under on-farm and *ex-situ* conservation were also analyzed. The number of specific alleles detected in the 2014 populations ranged from 2 (Nuogu2) to 55 (Paozhugu), accounting for 11.1–66.3 % of total alleles. The number of specific alleles detected in the 1980 populations ranged from 0 (Danuo) to 20 (Heigu), accounting for 0–50 % of total alleles. Except for Heinuo, Dalaogeng, Nuogu1, Nuogu2, Honggu, Heigu, Xihongmi and Danuo1, in the remaining 16 pairs, the number of specific alleles in the 2014 populations was 1.16–9.68 times that of the 1980 populations, and the frequency of specific alleles was higher in the 2014 populations than in the 1980 populations by 3.9–62.8 %. These results indicate that there was great variation in specific alleles in rice landraces collected from different times.

During the past 35 years of on-farm conservation, some alleles disappeared while some new ones appeared. Compared with the 1980 populations, 353 new specific alleles appeared, 204 specific alleles disappeared, a total of 559 alleles changed, and an average of 23.3 alleles per landrace changed. In 16 landrace pairs, there were more specific alleles in the 2014 populations than in the 1980 populations, comprising 67 % of total samples. In landraces under on-farm conservation, 1.2–55-fold more alleles appeared than disappeared. The proportion of newly appearing versus disappearing specific alleles was higher in the 2014 populations than in the 1980 populations. Paozhugu was the landrace with the most changes in specific alleles. In general, alleles from landraces under on-farm conservation were richer than those under *ex-situ* conservation, indicating that on-farm conservation promotes the maintenance and development of genetic diversity in rice landraces.

### Genetic differentiation of rice landraces between on-farm and *ex-situ* conservation

The results of AMOVA comparing populations under on-farm versus *ex-situ* conservation are shown in Table 4. The percentage of genetic variation of the 24 rice landrace pairs ranged from 1.23 % to 83.65 %, with all pairs showing highly significant differences (*P* < 0.001) between conservation practices except Danuo and Nuogu2. The differences between collection periods were the greatest for Bendidanuo, Xihongmi, Jiuyuenuo, Danuo1, Nuogu1, Honggu and Heigu, with values of more than 74 %. The variations in Lengshuigu, Huangnuogu, Xiangnuogu, Laogenghongjiao, Dibaigu, Dalaogeng, Lagengbaijiao, Danuo2, Xiaowuzui, Paozhugu and Yaduogu were large, ranging from 35 % to 70 %, while Nuogu2, Danuo, Heinuo, Huagu, Baixianghe and Ronghe had small differences of 1 % to 32 %, respectively. These results indicate that the genetic structures of rice landraces under on-farm conservation were significantly different from those under *ex-situ* conservation.

**Table 4:** Comparative genetic variations of on-farm and *ex-situ* conservation by AMOVA

Rice populations	Percentage of variation %		*P*	Rice populations	Percentage of variation %		*P*
	Between periods	Within periods			Between periods	Within periods	
Baixianghe	29.73	70.27	<0.001	Xiangnuogu	64.41	35.59	<0.001
Ronghe	31.15	68.85	<0.001	Nuogu1	76.80	23.20	<0.001
Danuo	2.69	97.31	0.070	Nuogu2	1.23	98.77	0.156
Heinuo	8.42	91.58	<0.001	Xiaowuzui	39.89	60.11	<0.001
Dalaogeng	60.65	39.35	<0.001	Dibaigu	61.10	38.90	<0.001
Huangnuogu	64.67	35.33	<0.001	Paozhugu	38.61	61.39	<0.001
Laogengbaijiao	59.62	40.38	<0.001	Bendidanuo	83.65	16.35	<0.001
Yaduogu	35.76	64.24	<0.001	Honggu	75.66	24.34	<0.001
Laogenghongjiao	63.83	36.17	<0.001	Heigu	74.38	25.62	<0.001
Huagu	17.78	82.22	<0.001	Xihongmi	83.64	16.36	<0.001
Lengshuigu	69.01	30.99	<0.001	Danuo1	77.19	22.81	<0.001
Jiuyuenuo	81.37	18.63	<0.001	Danuo2	59.48	40.52	<0.001

### Genetic phylogenetic tree of rice landraces from on-farm and *ex-situ* conservation

**Fig. 5:**
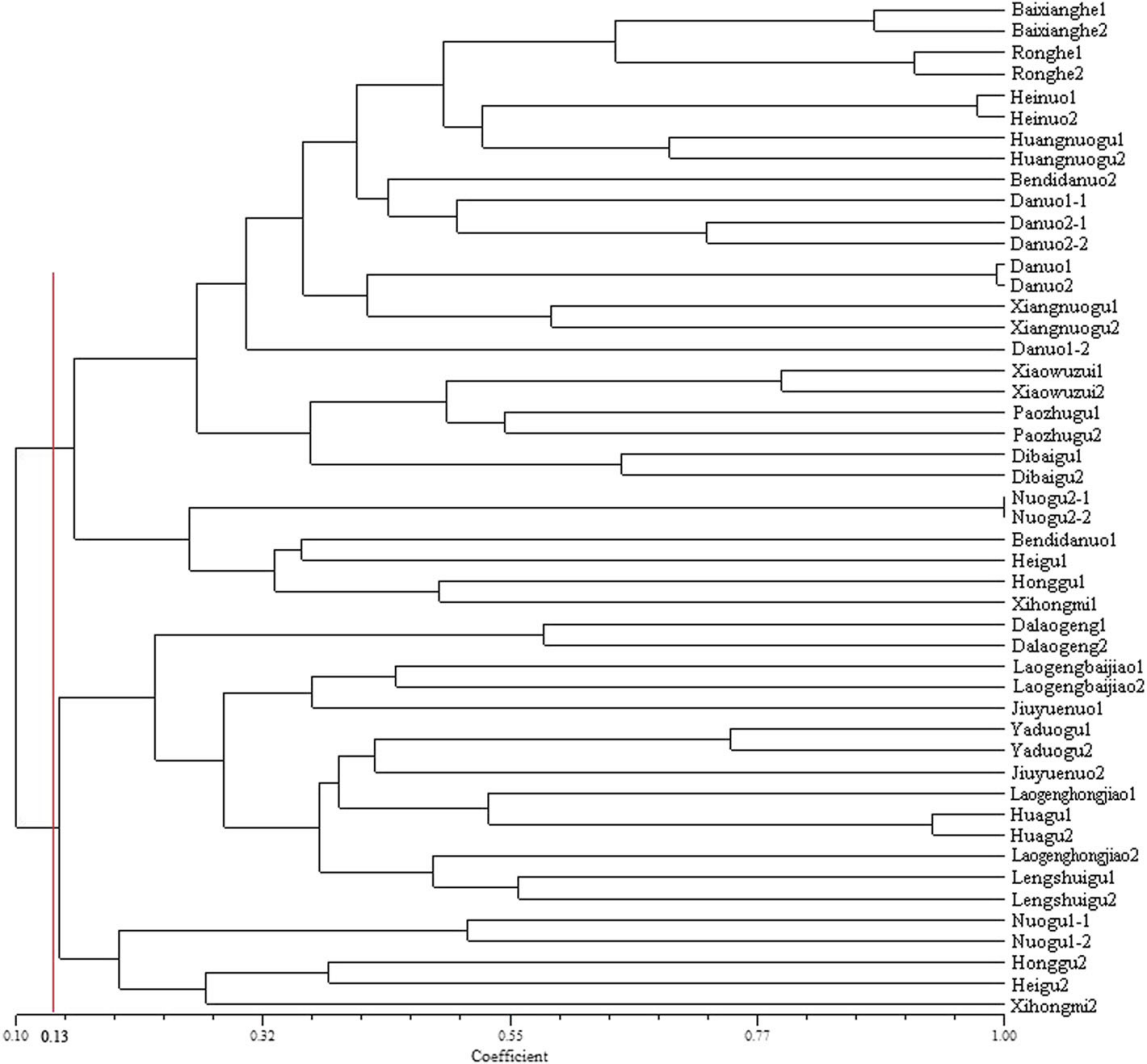
Dendrogram of the same rice landraces populations collected in different periods using SSR genetic coefficients

To identify the relationships among rice landraces under on-farm and *ex-situ* conservation, we classified 48 genotypes and constructed a phylogenetic tree using UPGMA cluster analysis (Fig. 5). In the phylogenetic tree, two major clusters were divided at a similarity coefficient level of 0.13. 13 rice landrace pairs and Honggu, Heigu, Xihongmi collected in 1980 from Hengxian county, Guangxi, were grouped in cluster I. Except for Nuogu2, Honggu, Heigu and Xihongmi, all of the remaining landraces (86.2 %) in cluster I were *Indica* type rice lines. 8 landrace pairs as well as Honggu, Heigu, Xihongmi collected in 2014 from small groups in cluster II. Except for Nuogu1 and Jiuyuenuo, all of the remaining landraces (78.9 %) in cluster II were *Japonica* type lines. 17 rice landrace pairs were contained in a small class, accounting for 71 % of pairs, with genetic similarity coefficient variation ranging from 0.4430 (Laogengbaijiao) to 0.9989 (Nuogu2). The 7 remaining pairs have genetic similarity coefficients of less than 0.3595. The results indicate that the degree of genetic variation of the same rice landrace differed under two conservation modes.

## Discussions

### Genetic diversity variation of rice landraces under on-farm and *ex-situ* conservation

We examined the changes in genetic diversity indexes (including Na, Ne, He and I) of the 24 rice landrace pairs under on-farm versus *ex-situ* conservation programs. More than half of the rice landrace pairs from on-farm populations had significantly more alleles and efficient alleles as well as higher He and I than the *ex-situ* populations. Specifically, 71 % of rice landrace pairs under on-farm conservation had higher Na than under *ex-situ* conservation, as well as 54 % of rice landraces pairs for Ne and 58 % pairs for both He and I. Moreover, the average genetic diversity index for the rice landraces was higher under on-farm conservation than under *ex-situ* conservation, the mean Na, Ne, He and I of the 2014 populations was 23.7 %, 12.3 %, 25 %, 30.4 %, higher than the 1980 populations separately. Overall, we observed higher genetic diversity under on-farm conservation than under *ex-situ* conditions. These results are consistent with the findings of Sun et al*.* [38], who investigated the genetic diversity of the same rice landraces under *in-situ* versus *ex-situ* conservation in Yunnan province, China.

The differences of genetic structure in rice landraces collected from farms and the National Gene Bank were marked. Of the 24 pairs examined, only Nuogu2 and Danuo had small genetic variation percentages and large genetic similarity coefficients, without significant differences. However, 22 pairs of rice landraces had, significant differences under on-farm versus *ex-situ* conservation, accounting for 92 % of lines. Phylogenetic analysis revealed that although 17 rice landrace pairs were present in a small cluster, the 15 pairs were significantly different except to Nuogu2 and Danuo. These results indicate that although the rice landrace pairs were selected in the same place and under similar environmental conditions, most of the same rice landraces conserved under on-farm and *ex-situ* programs were genetically distinct. Since each rice landrace pair collected from farms and the National Gene Bank had the same origin and growing conditions, as well as similar genetic structures, we therefore speculate that the variations in genetic structure between the same rice landraces under on-farm versus *ex-situ* conservation were mainly caused by the changes in local environmental and climatic conditions, and directional seed selection by local farmers during the process of long-term cultivation.

Previous comparative phenotypic and genetic diversity studies of crop landraces under on-farm and *ex-situ* protection had lead to similar conclusions through molecular markers and morphological analysis, respectively. Soleri & Smith [56] and Louette et al. [40], Tin et al. [42] and Barry et al. [44], Negri & Tiranti [26], Go ´mez et al. [43], Parzies et al. [41], Deu et al. [45] took use of corn from USA and Mexico, rice from Vietnam and Guinea, kidney beans from Italy, common bean from Nicaragua, barley from Syria, sorghum from Niger as materials, studied the genetic and morphological diversity within populations of local varieties preserved in Gene-Bank and farms. They found that on-farm conservation can maintain or enrich genetic heterogeneity and diversity. Analysis of the genetic diversity of rice landrace populations and wild rice populations using SSR molecular markers showed that populations under on-farm conservation typically have higher genetic diversity than those under *ex-situ* conservation [38, 57]. Traditional rice varieties of the Hani people from Yuanyang terrace in Yunnan province, such as Baigenglaojiao [58] (Gao et al. 2009) and Yuelianggu [59], were rich in genetic diversity when grown under on-farm conservation.

During the yearly process of rice cultivation by local farmers, landraces, which are genetically heterogeneous, continue to be subjected to the evolutionary pressures by the dual effects of natural environmental changes and farming cultural activities. The results showed that the origin of 12.3–30.4 % of higher richness of alleles in 35 years. Environmental pressures may lead to gene mutation, recombination and drift in rice landraces, but the ways of seed selection and seed management are particularly important. One hand, farmers and neighbors from the same or different villages established the nets of interchange of seeds, exchanged their own seed with others in farming and culture activities. For instance, Dong and Dai people exchanged rice seeds through taking seeds as gifts in the wedding ceremony. The other hand, the local government of ethnic groups may release of new varieties from seed banks, which lead to gene flow, interactions among landraces, and probably with wild relatives, the hybrid rice had been cultivating in majority regions of China for more than 40 years and had been produced a profound influence on rice landraces. Genetic variation of rice landraces may represent positive variation, but it may also represent negative variation. Therefore, positive and negative genetic variations appear simultaneously. Positive mutations would be preserved, while negative variation would be eliminated during natural selection and plantation. Therefore, on-farm conservation of rice landraces not only enriches genetic diversity and allelic variation within populations, but it also helps maintaining resistance genes and other beneficial genes for rice breeding and production, promoting the protection of rice genetic resources as well as traditional cultural practices. Influence of ethnic traditional cultures in the higher genetic diversity of rice landraces under on-farm conservation versus *ex-situ* conservation.

In this study, we found that the genetic diversity of rice landraces within populations was greater under on-farm conservation than under *ex-situ* conservation, especially the diversity of alleles, which may be closely related to specific natural environmental conditions and traditional practices of minority people. The 24 rice landrace pairs were obtained from ethnic minority areas in Guizhou, Yunnan and Guangxi provinces, which are rich in traditional ethnic farming practices and have complicated environmental conditions. These rice landraces resources under long-term cultivation that are used by local farmers, which are associated with traditional ethnic customs and play a crucial role in the livelihood of the local farmers. During the process of seed preservation and on-farm conservation, the adaptive capacities of rice landraces to the environment, variety evolution and improvement are constantly promoted. 4 rice landrace pairs from Guizhou province are traditional glutinous varieties used by the Dong and Miao people. 14 traditional varieties were obtained from Yunnan province, including 9 red and glutinous rice landraces from Yuanyang terrace inhabited by the Hani minority and 5 glutinous pairs from Xishuangbanna tropical regions of the Dai and Bulang people. 6 rice variety pairs were inhabited by the Zhuang, Yao and Maonan in the Guangxi Zhuang Autonomous Region.

The rice landraces under on-farm conservation, which were preserved and planted in farms, are indispensable to daily life and the traditional cultural activities of local ethnic groups. For instance, traditional rice landraces including Kam Sweet Rice, glutinous and red rice are associated with the food cultures, festivals and religious beliefs of the Dong, Hani and Zhuang ethnic groups. On the other hand, different rice landraces grow in complex and versatile environments, such as various climate types including tropical, subtropical and temperate climates; various landforms including river valleys, basins, hills, mountains and staggered plateaus; and ever-changing precipitation levels, light intensity, temperatures and other meteorological factors. In general, during the process of on-farm conservation, the complex ecological and climatic conditions, traditional farming practices and cultural customs of different ethnic groups might promote genetic variation of rice landraces, thereby having a positive effect on genetic diversity within a population.

Many studies examining the relationship between ethnic minority cultures and genetic diversity of traditional landraces have come to similar conclusions about the Yunnan province contains the most concentrated areas of ethnic minorities and traditional cultures in China. The Hani, Dai and Wa peoples maintained many varieties of rice, wheat and maize, which are uniquely adapted to both highly heterogeneous agro-ecological conditions and cultural needs [60, 61]. Lei et al. [62] and Wang et al. [63] found that Kam Sweet Rice, the traditional rice landrace of Dong people, has been well preserved for thousands of years in southeast Guizhou province, as it meets the needs of local ethnic customs and traditional cultures.

### Suggestions for effective genetic diversity conservation of rice landraces

Previous studies have shown that on-farm conservation can be used as a supplementary approach to *ex-situ* protection, as this management process promotes the dynamic evolution, adaptation and preservation of the genetic diversity of crop varieties. The advantages of on-farm conservation and the feasibility of its implementation were well recognized in the Himalayan region of India [64, 65], France [66] and Turkey [67]. In addition, Diwakar et al. [68] found that farmers were willing to pay more attention to on-farm conservation, which was beneficial for their direct economic interests. The current results show that rice landraces under on-farm conservation produced more alleles and exhibited an enriched genetic background compared to those under *ex-situ* conservation. Thus, on-farm conservation is superior to *ex-situ* conservation for maintaining and increasing the genetic diversity of rice landraces while avoiding the loss of favorable genes. This finding indicates that it is necessary to implement on-farm conservation in the ethnic areas of Yunnan, Guizhou and Guangxi. We suggest that conservation demonstration areas should be established in these regions, and could be carried out at the cultural, social, economic and scientific level to protect the genetic diversity of rice landraces.

As mentioned above, the rice landraces used in this experiment have been cultivated and used by ethnic groups for hundreds or even thousands of years, which were preserved to meet the nutritional, cultural and religious needs of the local people. Many farmers of the Zhuang, Yao, Maonan and Dai nationalities reserve very small areas of farmland to plant glutinous rice landraces to be used for ethnic festivals, sacrifices to ancestors, weddings and funerals. The cultivation of traditional rice varieties represents an important aspect of local cultures. Therefore, promoting on-farm conservation of rice landraces indirectly protects traditional ethnic cultures. Firstly, in order to protect rice genetic resources and increase the enthusiasm of farmers for growing and using rice landraces, it is necessary for the elderly to teach young people to continue to grow rice landraces and to realize the cultural value of rice landraces. At the same time, as there are a large number of young migrant workers, the government should invest in providing young people with more employment opportunities in their hometowns, thereby facilitating the maintenance of traditional farming practices of rice landraces via on-farm conservation. Secondly, more people should be encouraged to participate in the conservation of traditional variety resources by promoting awareness of traditional crop varieties, establishing rural seed banks and carrying out seed exchange and cultural exchange activities. Thirdly, the government should support the commercial cultivation and development of agricultural products based on rice landraces and should also increase the market price of traditional rice varieties, which would be beneficial to farmers while promoting the implementation of on-farm protection. Since 2007, a rice company from Guizhou province has been employing the “company + cooperative + household” mode to develop and market organic products made from Kam Sweet Rice, the glutinous rice landrace of the Dong people, which has significantly increased the incomes of farmers and effectively conserved rice germplasm resources.

Finally, scientific research institutions should establish gene banks for rice landraces and other traditional germplasm resources at the national, local or community level, which could serve as a supplementary method to on-farm conservation (Additional file 1). As we determined that the genetic structure and diversity of rice landraces changed significantly under on-farm conservation, it is necessary to re-collect rice landraces at regular intervals to protect their genetic diversity and integrity. However, identifying the optimal intervals for collection requires further study. In addition, Participatory Plant Breeding (PPB) projects should be supported by local governments and research institutions in the community. Scientists and local farmers should work together to select favorable rice varieties from traditional landraces through conventional breeding approaches. Such cooperation would not only improve the quality of traditional varieties and increase their productivity, it would also increase the knowledge level of farmers and enhance the effectiveness of on-farm conservation. For example, farmers in Nepal involved in PPB projects have successfully sold seeds of new varieties at higher prices than those of old landraces [69]. In addition, Yao people from Guangxi province have upgraded traditional maize landrace cultivation practices through PPB projects, producing crops with higher yields and quality than traditional varieties, which has both social and economic benefits.

## Conclusion

Most China’s rice landrace varieties were preserved or saved in the ethnic areas of southwest China. Compared with the *ex-situ* conservation approaches, rice landraces under on-farm conservation programs in Guizhou, Yunnan and Guangxi provinces had more alleles and higher genetic diversity over the past 35 years. On-farm conservation can effectively promote the allelic variation and increase the genetic diversity of rice landraces. In every site we investigated, we found extensive traditional cultures on rice landraces and its management. Ethnic traditional cultures have not only protected the rice landrace germplasm resources on-farm, but also improved the genetic diversity of rice landraces. Moreover, this study elucidated that ethnic traditional cultures and custom practices are crucial foundations to implement on-farm conservation.

## Additional file

Additional file 1: Supplementary materials. (DOCX 62 kb)

## Abbreviations

AMOVA: Analysis of molecular variance
He: Nei’s genetic diversity index
I: Shannon’s information index
Na: Number of alleles
Ne: Effective number of alleles
PPB: Participatory plant breeding
SSR: Microsatellite or simple sequence repeat
UPGMA: Unweighted pair group method with arithmetic
