# Supplementary material for: Influence of ethnic traditional cultures on genetic diversity of rice landraces under on-farm conservation in southwest China
Source: J Ethnobiol Ethnomed. 2016 Oct 27;12:51. doi: 10.1186/s13002-016-0120-0 (PMC5084377; doi:10.1186/s13002-016-0120-0)
Supplement: Supplementary file 1 — Supplementary materials. (DOCX 62 kb) [file 13002_2016_120_MOESM1_ESM.docx]

**Supplementary materials:**

**(1) 13 pairs of microsatellite markers information are shown in Tab.1**

**Tab.1 SSR markers information**

| **Locus** | **Chromosomes** | **Forward primer** | **Reverse primer** | **Position/cM** | **pp** |
| --- | --- | --- | --- | --- | --- |
| RM449 | 1 | ttgggaggtgttgataaggc | accaccagcgtctctctctc | 73.1 | 116-138 |
| RM48 | 2 | tgtcccactgctttcaagc | cgagaatgagggacaaataacc | 190.2 | 199-221 |
| RM251 | 3 | gaatggcaatggcgctag | atgcggttcaagattcgatc | 79.1 | 114-150 |
| RM471 | 4 | acgcacaagcagatgatgag | gggagaagacgaatgtttgc | 53.8 | 108-120 |
| RM241 | 4 | gagccaaataagatcgctga | tgcaagcagcagatttagtg | 106.2 | 102-142 |
| RM586 | 6 | acctcgcgttattaggtaccc | gagatacgccaacgagatacc | 7.4 | 241-281 |
| RM180 | 7 | ctacatcggcttaggtgtagcaacacg | acttgctctacttgtggtgagggactg | 30.1 | 107-204 |
| RM336 | 7 | cttacagagaaacggcatcg | gctggtttgtttcaggttcg | 61.00 | 148-193 |
| RM223 | 8 | gagtgagcttgggctgaaac | gaaggcaagtcttggcactg | 75.7 | 139-163 |
| RM257 | 9 | cagttccgagcaagagtactc | ggatcggacgtggcatatg | 66.1 | 121-173 |
| RM333 | 10 | gtacgactacgagtgtcaccaa | gtcttcgcgatcactcgc | 110.4 | 164-215 |
| RM287 | 11 | ttccctgttaagagagaaatc | gtgtatttggtgaaagcaac | 68.6 | 98-118 |
| RM247 | 12 | tagtgccgatcgatgtaacg | catatggttttgacaaagcg | 32.3 | 130-176 |

**(2) The results of genetic diversity indexes including Na, Ne, He, and I are shown in Tab.2-Tab.5.**

Tab.2 Number of alleles (Na) for the rice landraces collected in different periods

| Locus | **Number of alleles (Na)** | | | | | | | | | | | | | | | | | | | | | | | |
| --- | --- | --- | --- | --- | --- | --- | --- | --- | --- | --- | --- | --- | --- | --- | --- | --- | --- | --- | --- | --- | --- | --- | --- | --- |
|  | Baixianghe | | Ronghe | | Danuo | | Heinuo | | Dalaogeng | | Huangnuogu | | Laogengbaijiao | | Yaduogu | | Laogenghongjiao | | Huagu | | Lengshuigu | | Jiuyuenuo | |
|  | 2014 | 1980 | 2014 | 1980 | 2014 | 1980 | 2014 | 1980 | 2014 | 1980 | 2014 | 1980 | 2014 | 1980 | 2014 | 1980 | 2014 | 1980 | 2014 | 1980 | 2014 | 1980 | 2014 | 1980 |
| RM449 | 2 | 1 | 1 | 1 | 2 | 1 | 1 | 2 | 2 | 1 | 2 | 2 | 3 | 1 | 1 | 1 | 1 | 1 | 3 | 2 | 2 | 1 | 1 | 1 |
| RM48 | 3 | 1 | 2 | 1 | 1 | 1 | 1 | 2 | 2 | 1 | 1 | 1 | 2 | 4 | 2 | 7 | 3 | 2 | 4 | 3 | 2 | 2 | 4 | 2 |
| RM251 | 3 | 1 | 2 | 2 | 1 | 1 | 1 | 1 | 2 | 2 | 1 | 1 | 4 | 4 | 4 | 3 | 5 | 4 | 7 | 5 | 3 | 3 | 3 | 3 |
| RM471 | 3 | 1 | 2 | 3 | 1 | 1 | 1 | 1 | 3 | 3 | 2 | 1 | 3 | 2 | 2 | 2 | 2 | 1 | 2 | 2 | 3 | 1 | 1 | 1 |
| RM241 | 4 | 2 | 3 | 2 | 2 | 2 | 2 | 3 | 3 | 3 | 2 | 1 | 3 | 4 | 3 | 2 | 2 | 2 | 6 | 4 | 3 | 2 | 4 | 1 |
| RM586 | 3 | 3 | 4 | 4 | 3 | 3 | 4 | 4 | 3 | 3 | 2 | 3 | 2 | 2 | 2 | 1 | 3 | 1 | 4 | 1 | 2 | 1 | 2 | 3 |
| RM336 | 5 | 4 | 3 | 2 | 4 | 3 | 3 | 2 | 1 | 3 | 2 | 2 | 3 | 6 | 2 | 2 | 3 | 3 | 3 | 3 | 2 | 2 | 3 | 1 |
| RM180 | 1 | 2 | 2 | 2 | 1 | 1 | 2 | 2 | 3 | 3 | 2 | 2 | 3 | 3 | 4 | 3 | 3 | 3 | 1 | 1 | 4 | 3 | 2 | 1 |
| RM223 | 2 | 2 | 1 | 1 | 1 | 1 | 2 | 2 | 1 | 3 | 3 | 2 | 3 | 2 | 3 | 2 | 4 | 3 | 3 | 2 | 5 | 1 | 2 | 2 |
| RM257 | 2 | 2 | 2 | 3 | 2 | 2 | 2 | 3 | 2 | 1 | 2 | 2 | 4 | 3 | 3 | 3 | 2 | 2 | 4 | 5 | 5 | 2 | 2 | 1 |
| RM333 | 6 | 3 | 3 | 1 | 7 | 6 | 2 | 2 | 5 | 4 | 3 | 1 | 5 | 2 | 9 | 5 | 3 | 3 | 3 | 4 | 4 | 3 | 1 | 2 |
| RM287 | 2 | 1 | 1 | 1 | 1 | 1 | 1 | 1 | 1 | 2 | 3 | 3 | 3 | 2 | 2 | 3 | 2 | 2 | 2 | 2 | 3 | 2 | 2 | 1 |
| RM247 | 2 | 1 | 3 | 1 | 2 | 2 | 2 | 1 | 1 | 2 | 3 | 2 | 3 | 2 | 2 | 3 | 5 | 3 | 4 | 3 | 3 | 2 | 5 | 2 |
| **总计Total** | **28** | **19** | **22** | **21** | **18** | **16** | **19** | **22** | **22** | **23** | **19** | **17** | **30** | **31** | **26** | **26** | **28** | **22** | **37** | **28** | **31** | **18** | **24** | **16** |
| **平均Mean** | **2.92** | **1.85** | **2.23** | **1.85** | **2.15** | **1.92** | **1.85** | **2.00** | **2.23** | **2.38** | **2.15** | **1.77** | **3.15** | **2.85** | **3.00** | **2.85** | **2.92** | **2.31** | **3.54** | **2.85** | **3.15** | **1.38** | **2.46** | **1.62** |

（continued）Tab.2 Number of alleles (Na) for the rice landraces collected in different periods

| Locus | **Number of alleles (Na)** | | | | | | | | | | | | | | | | | | | | | | | |
| --- | --- | --- | --- | --- | --- | --- | --- | --- | --- | --- | --- | --- | --- | --- | --- | --- | --- | --- | --- | --- | --- | --- | --- | --- |
|  | Xiangnuogu | | Nuogu1 | | Nuogu2 | | Xiaowuzui | | Dibaigu | | Paozhugu | | Bendidanuo | | Honggu | | Heigu | | Xihongmi | | Danuo1 | | Danuo2 | |
|  | 2014 | 1980 | 2014 | 1980 | 2014 | 1980 | 2014 | 1980 | 2014 | 1980 | 2014 | 1980 | 2014 | 1980 | 2014 | 1980 | 2014 | 1980 | 2014 | 1980 | 2014 | 1980 | 2014 | 1980 |
| RM449 | 2 | 1 | 3 | 3 | 1 | 2 | 2 | 2 | 1 | 2 | 4 | 2 | 2 | 1 | 3 | 2 | 2 | 3 | 1 | 1 | 1 | 1 | 1 | 1 |
| RM48 | 2 | 3 | 3 | 3 | 2 | 1 | 3 | 3 | 2 | 1 | 5 | 1 | 3 | 1 | 2 | 3 | 1 | 2 | 1 | 1 | 3 | 2 | 2 | 1 |
| RM251 | 1 | 1 | 4 | 4 | 1 | 2 | 2 | 2 | 1 | 1 | 12 | 2 | 4 | 1 | 1 | 1 | 2 | 5 | 3 | 1 | 1 | 2 | 2 | 1 |
| RM471 | 1 | 1 | 2 | 2 | 1 | 1 | 1 | 2 | 2 | 1 | 6 | 3 | 2 | 1 | 1 | 1 | 1 | 3 | 1 | 1 | 2 | 2 | 1 | 2 |
| RM241 | 5 | 2 | 3 | 3 | 1 | 2 | 6 | 3 | 5 | 3 | 9 | 3 | 3 | 2 | 2 | 5 | 2 | 4 | 1 | 1 | 1 | 1 | 4 | 3 |
| RM586 | 3 | 2 | 2 | 2 | 1 | 2 | 2 | 1 | 3 | 3 | 8 | 3 | 2 | 1 | 2 | 4 | 4 | 4 | 2 | 2 | 2 | 2 | 3 | 1 |
| RM336 | 3 | 1 | 2 | 3 | 1 | 1 | 1 | 1 | 3 | 1 | 7 | 2 | 3 | 2 | 4 | 2 | 2 | 3 | 2 | 2 | 3 | 4 | 2 | 3 |
| RM180 | 3 | 4 | 2 | 3 | 1 | 1 | 2 | 1 | 2 | 2 | 5 | 2 | 3 | 2 | 2 | 2 | 1 | 2 | 2 | 2 | 2 | 1 | 2 | 1 |
| RM223 | 2 | 2 | 4 | 5 | 3 | 2 | 3 | 1 | 3 | 1 | 4 | 2 | 3 | 3 | 3 | 2 | 4 | 3 | 1 | 2 | 2 | 2 | 2 | 1 |
| RM257 | 2 | 3 | 2 | 3 | 2 | 2 | 4 | 4 | 8 | 6 | 5 | 2 | 4 | 1 | 2 | 3 | 2 | 3 | 1 | 2 | 3 | 2 | 2 | 2 |
| RM333 | 4 | 2 | 3 | 4 | 2 | 2 | 5 | 1 | 4 | 4 | 5 | 3 | 4 | 6 | 2 | 2 | 1 | 1 | 3 | 4 | 1 | 3 | 2 | 1 |
| RM287 | 1 | 1 | 4 | 3 | 1 | 2 | 3 | 1 | 1 | 1 | 8 | 3 | 4 | 1 | 3 | 3 | 5 | 4 | 3 | 3 | 1 | 1 | 1 | 1 |
| RM247 | 2 | 4 | 2 | 2 | 1 | 2 | 5 | 4 | 4 | 3 | 6 | 2 | 3 | 1 | 1 | 1 | 2 | 3 | 3 | 2 | 2 | 3 | 1 | 1 |
| **总计Total** | **24** | **20** | **27** | **31** | **14** | **16** | **26** | **20** | **30** | **21** | **65** | **22** | **29** | **15** | **22** | **25** | **21** | **32** | **15** | **15** | **20** | **19** | **21** | **16** |
| **平均Mean** | **2.38** | **2.08** | **2.77** | **3.08** | **1.38** | **1.69** | **3.00** | **2.00** | **3.00** | **2.23** | **6.46** | **2.31** | **3.08** | **1.77** | **2.15** | **2.38** | **2.23** | **3.08** | **1.85** | **1.85** | **1.85** | **2.00** | **1.92** | **1.46** |

Tab.3 Effective number of alleles (Ne) for the rice landraces collected in different periods

| Locus | **Effective number of alleles（Ne）** | | | | | | | | | | | | | | | | | | | | | | | |
| --- | --- | --- | --- | --- | --- | --- | --- | --- | --- | --- | --- | --- | --- | --- | --- | --- | --- | --- | --- | --- | --- | --- | --- | --- |
|  | Baixianghe | | Ronghe | | Danuo | | Heinuo | | Dalaogeng | | Huangnuogu | | Laogengbaijiao | | Yaduogu | | Laogenghongjiao | | Huagu | | Lengshuigu | | Jiuyuenuo | |
|  | 2014 | 1980 | 2014 | 1980 | 2014 | 1980 | 2014 | 1980 | 2014 | 1980 | 2014 | 1980 | 2014 | 1980 | 2014 | 1980 | 2014 | 1980 | 2014 | 1980 | 2014 | 1980 | 2014 | 1980 |
| RM449 | 1.210 | 1.000 | 1.000 | 1.000 | 1.044 | 1.000 | 1.000 | 1.117 | 1.021 | 1.000 | 1.394 | 1.043 | 1.065 | 1.000 | 1.000 | 1.000 | 1.000 | 1.000 | 1.176 | 1.139 | 1.043 | 1.000 | 1.000 | 1.000 |
| RM48 | 1.215 | 1.000 | 1.089 | 1.000 | 1.000 | 1.000 | 1.000 | 1.045 | 1.043 | 1.000 | 1.000 | 1.000 | 1.332 | 2.126 | 1.280 | 2.304 | 1.341 | 1.133 | 1.130 | 1.301 | 1.653 | 1.180 | 1.090 | 1.043 |
| RM251 | 1.170 | 1.000 | 1.944 | 1.642 | 1.000 | 1.000 | 1.000 | 1.000 | 1.043 | 1.492 | 1.000 | 1.000 | 1.667 | 1.923 | 2.395 | 1.396 | 1.355 | 1.440 | 1.741 | 3.893 | 1.265 | 1.135 | 1.188 | 1.410 |
| RM471 | 1.346 | 1.000 | 1.209 | 1.225 | 1.000 | 1.000 | 1.000 | 1.000 | 1.720 | 1.159 | 1.089 | 1.000 | 1.811 | 1.133 | 1.043 | 1.087 | 1.133 | 1.000 | 1.083 | 1.044 | 1.088 | 1.000 | 1.000 | 1.000 |
| RM241 | 1.617 | 1.686 | 2.625 | 1.471 | 1.091 | 1.293 | 1.280 | 1.672 | 1.185 | 1.111 | 1.089 | 1.000 | 1.401 | 2.650 | 1.351 | 1.087 | 1.205 | 1.180 | 2.641 | 2.466 | 2.076 | 1.180 | 1.246 | 1.000 |
| RM586 | 2.064 | 1.865 | 1.834 | 1.475 | 1.723 | 1.948 | 2.969 | 2.600 | 1.682 | 1.471 | 1.044 | 2.875 | 1.332 | 1.043 | 1.704 | 1.000 | 2.612 | 1.000 | 1.229 | 1.000 | 1.043 | 1.000 | 1.287 | 2.165 |
| RM336 | 1.441 | 1.933 | 1.521 | 1.142 | 1.423 | 1.092 | 1.957 | 1.991 | 1.000 | 1.185 | 1.999 | 1.043 | 1.210 | 2.082 | 1.043 | 1.627 | 1.088 | 1.261 | 1.084 | 1.271 | 1.087 | 1.043 | 1.138 | 1.000 |
| RM180 | 1.000 | 1.996 | 1.044 | 1.023 | 1.000 | 1.000 | 1.653 | 1.642 | 2.026 | 1.769 | 1.160 | 1.180 | 2.264 | 1.261 | 1.417 | 1.043 | 1.837 | 1.088 | 1.000 | 1.000 | 1.239 | 1.159 | 1.044 | 1.000 |
| RM223 | 1.304 | 1.047 | 1.000 | 1.000 | 1.000 | 1.000 | 1.043 | 1.045 | 1.000 | 2.039 | 1.623 | 1.087 | 1.585 | 1.043 | 1.396 | 1.438 | 1.957 | 1.341 | 1.274 | 1.516 | 1.525 | 1.000 | 1.394 | 1.230 |
| RM257 | 1.166 | 1.198 | 1.112 | 1.198 | 1.923 | 1.941 | 1.385 | 1.367 | 1.043 | 1.000 | 1.136 | 1.133 | 1.293 | 2.129 | 2.723 | 2.327 | 1.043 | 1.043 | 1.459 | 1.587 | 1.540 | 1.043 | 1.394 | 1.000 |
| RM333 | 3.962 | 2.367 | 1.090 | 1.000 | 2.311 | 2.453 | 1.704 | 1.976 | 2.678 | 2.228 | 1.774 | 1.000 | 2.361 | 1.043 | 6.054 | 3.408 | 1.341 | 1.453 | 1.274 | 1.311 | 1.786 | 1.287 | 1.000 | 1.043 |
| RM287 | 1.210 | 1.000 | 1.000 | 1.000 | 1.000 | 1.000 | 1.000 | 1.000 | 1.000 | 1.600 | 1.114 | 1.185 | 1.135 | 1.043 | 1.044 | 1.963 | 1.573 | 1.230 | 1.127 | 1.091 | 1.341 | 1.043 | 1.340 | 1.000 |
| RM247 | 1.210 | 1.000 | 1.044 | 1.000 | 1.734 | 1.996 | 1.043 | 1.000 | 1.000 | 1.546 | 1.356 | 1.043 | 1.088 | 1.087 | 1.546 | 2.153 | 2.468 | 1.464 | 1.228 | 1.246 | 1.411 | 1.180 | 1.306 | 1.332 |
| **总计Total** | **19.915** | **18.091** | **17.511** | **15.175** | **17.250** | **17.724** | **18.032** | **18.456** | **17.439** | **18.601** | **16.776** | **15.587** | **19.543** | **19.561** | **23.996** | **21.834** | **19.953** | **15.631** | **17.447** | **19.865** | **18.096** | **14.250** | **15.426** | **15.222** |
| **平均Mean** | **1.532** | **1.392** | **1.347** | **1.167** | **1.327** | **1.363** | **1.387** | **1.420** | **1.341** | **1.431** | **1.291** | **1.199** | **1.503** | **1.505** | **1.846** | **1.680** | **1.535** | **1.202** | **1.342** | **1.528** | **1.392** | **1.096** | **1.187** | **1.171** |

(continued) Tab.3 Effective number of alleles (Ne) for the rice landraces collected in different periods

| Locus | **Effective number of alleles（Ne）** | | | | | | | | | | | | | | | | | | | | | | | |
| --- | --- | --- | --- | --- | --- | --- | --- | --- | --- | --- | --- | --- | --- | --- | --- | --- | --- | --- | --- | --- | --- | --- | --- | --- |
|  | Xiangnuogu | | Nuogu1 | | Nuogu2 | | Xiaowuzui | | Dibaigu | | Paozhugu | | Bendidanuo | | Honggu | | Heigu | | Xihongmi | | Danuo1 | | Danuo2 | |
|  | 2014 | 1980 | 2014 | 1980 | 2014 | 1980 | 2014 | 1980 | 2014 | 1980 | 2014 | 1980 | 2014 | 1980 | 2014 | 1980 | 2014 | 1980 | 2014 | 1980 | 2014 | 1980 | 2014 | 1980 |
| RM449 | 1.133 | 1.000 | 1.088 | 1.111 | 1.000 | 1.087 | 1.822 | 1.043 | 1.000 | 1.043 | 2.095 | 1.385 | 1.044 | 1.000 | 1.088 | 1.385 | 1.043 | 1.646 | 1.000 | 1.000 | 1.000 | 1.000 | 1.000 | 1.000 |
| RM48 | 1.043 | 1.088 | 1.088 | 1.235 | 1.087 | 1.000 | 1.606 | 1.235 | 1.546 | 1.000 | 2.823 | 1.000 | 1.090 | 1.000 | 1.043 | 1.573 | 1.000 | 1.087 | 1.000 | 1.000 | 1.292 | 1.230 | 1.600 | 1.000 |
| RM251 | 1.000 | 1.000 | 1.186 | 1.264 | 1.000 | 1.064 | 1.043 | 1.043 | 1.000 | 1.000 | 4.997 | 1.504 | 1.191 | 1.000 | 1.000 | 1.000 | 1.043 | 1.985 | 1.135 | 1.000 | 1.000 | 1.280 | 1.800 | 1.000 |
| RM471 | 1.000 | 1.000 | 1.043 | 1.043 | 1.000 | 1.000 | 1.000 | 1.043 | 1.180 | 1.000 | 2.182 | 1.539 | 1.044 | 1.000 | 1.000 | 1.000 | 1.000 | 1.235 | 1.000 | 1.000 | 1.546 | 1.180 | 1.000 | 1.043 |
| RM241 | 2.521 | 1.180 | 1.111 | 1.184 | 1.000 | 1.021 | 2.723 | 1.235 | 2.411 | 2.133 | 5.961 | 1.235 | 1.090 | 1.087 | 1.997 | 3.105 | 1.133 | 1.697 | 1.000 | 1.000 | 1.000 | 1.000 | 2.340 | 1.185 |
| RM586 | 1.453 | 1.546 | 1.043 | 1.087 | 1.000 | 1.064 | 1.280 | 1.000 | 1.065 | 1.065 | 5.322 | 1.184 | 1.044 | 1.000 | 1.043 | 1.548 | 1.136 | 1.732 | 1.064 | 1.704 | 1.180 | 1.230 | 1.557 | 1.000 |
| RM336 | 1.135 | 1.000 | 1.021 | 1.184 | 1.000 | 1.000 | 1.000 | 1.000 | 1.135 | 1.000 | 4.546 | 1.492 | 1.044 | 1.087 | 2.195 | 1.332 | 1.332 | 1.412 | 1.205 | 1.021 | 1.540 | 2.025 | 1.753 | 2.061 |
| RM180 | 2.723 | 2.358 | 1.043 | 1.185 | 1.000 | 1.000 | 1.979 | 1.000 | 1.255 | 1.205 | 1.143 | 1.306 | 1.066 | 1.280 | 1.087 | 1.332 | 1.000 | 1.492 | 1.986 | 1.230 | 1.332 | 1.000 | 1.180 | 1.000 |
| RM223 | 1.087 | 1.230 | 1.290 | 1.391 | 2.026 | 1.986 | 1.625 | 1.000 | 1.182 | 1.000 | 3.030 | 1.043 | 2.886 | 1.235 | 1.753 | 1.438 | 1.725 | 1.669 | 1.000 | 1.332 | 1.043 | 1.492 | 1.900 | 1.000 |
| RM257 | 1.043 | 1.997 | 1.087 | 1.184 | 1.043 | 1.280 | 1.923 | 1.958 | 3.879 | 1.300 | 1.974 | 1.986 | 1.216 | 1.000 | 1.043 | 1.662 | 1.043 | 1.753 | 1.000 | 1.087 | 1.290 | 1.087 | 1.385 | 1.087 |
| RM333 | 1.732 | 1.306 | 1.135 | 1.240 | 1.043 | 1.087 | 2.824 | 1.000 | 1.843 | 2.140 | 1.921 | 1.868 | 1.164 | 1.528 | 1.230 | 1.043 | 1.000 | 1.000 | 1.088 | 2.144 | 1.000 | 1.759 | 1.133 | 1.000 |
| RM287 | 1.000 | 1.000 | 1.532 | 1.235 | 1.000 | 1.043 | 1.184 | 1.000 | 1.000 | 1.000 | 5.620 | 1.065 | 1.966 | 1.000 | 1.088 | 1.287 | 1.298 | 1.266 | 1.088 | 1.615 | 1.000 | 1.000 | 1.000 | 1.000 |
| RM247 | 1.043 | 1.421 | 1.043 | 1.043 | 1.000 | 1.064 | 3.133 | 2.952 | 1.296 | 1.088 | 3.868 | 1.230 | 1.522 | 1.000 | 1.000 | 1.000 | 1.087 | 1.088 | 1.088 | 1.043 | 1.205 | 1.287 | 1.000 | 1.000 |
| **总计Total** | **17.911** | **17.124** | **14.708** | **15.384** | **14.197** | **14.697** | **23.141** | **16.506** | **19.793** | **15.974** | **45.481** | **17.835** | **17.366** | **14.217** | **16.564** | **18.705** | **14.838** | **19.062** | **14.654** | **16.175** | **15.427** | **16.569** | **18.649** | **14.375** |
| **平均Mean** | **1.378** | **1.317** | **1.131** | **1.183** | **1.092** | **1.131** | **1.780** | **1.270** | **1.523** | **1.229** | **3.499** | **1.372** | **1.336** | **1.094** | **1.274** | **1.439** | **1.141** | **1.466** | **1.127** | **1.244** | **1.187** | **1.275** | **1.435** | **1.106** |

Tab.4 Nei’s genetic diversity index (He) for the rice landraces collected in different periods

| Locus | **Nei's genetic diversity index (He)** | | | | | | | | | | | | | | | | | | | | | | | |
| --- | --- | --- | --- | --- | --- | --- | --- | --- | --- | --- | --- | --- | --- | --- | --- | --- | --- | --- | --- | --- | --- | --- | --- | --- |
|  | Baixianghe | | Ronghe | | Danuo | | Heinuo | | Dalaogeng | | Huangnuogu | | Laogengbaijiao | | Yaduogu | | Laogenghongjiao | | Huagu | | Lengshuigu | | Jiuyuenuo | |
|  | 2014 | 1980 | 2014 | 1980 | 2014 | 1980 | 2014 | 1980 | 2014 | 1980 | 2014 | 1980 | 2014 | 1980 | 2014 | 1980 | 2014 | 1980 | 2014 | 1980 | 2014 | 1980 | 2014 | 1980 |
| RM449 | 0.174 | 0.000 | 0.000 | 0.000 | 0.043 | 0.000 | 0.000 | 0.105 | 0.021 | 0.000 | 0.283 | 0.041 | 0.061 | 0.000 | 0.000 | 0.000 | 0.000 | 0.000 | 0.150 | 0.122 | 0.041 | 0.000 | 0.000 | 0.000 |
| RM48 | 0.177 | 0.000 | 0.082 | 0.000 | 0.000 | 0.000 | 0.000 | 0.044 | 0.041 | 0.000 | 0.000 | 0.000 | 0.249 | 0.530 | 0.219 | 0.566 | 0.254 | 0.117 | 0.115 | 0.232 | 0.395 | 0.153 | 0.083 | 0.041 |
| RM251 | 0.145 | 0.000 | 0.486 | 0.391 | 0.000 | 0.000 | 0.000 | 0.000 | 0.041 | 0.330 | 0.000 | 0.000 | 0.400 | 0.480 | 0.583 | 0.284 | 0.262 | 0.305 | 0.426 | 0.743 | 0.209 | 0.119 | 0.158 | 0.291 |
| RM471 | 0.257 | 0.000 | 0.173 | 0.184 | 0.000 | 0.000 | 0.000 | 0.000 | 0.419 | 0.137 | 0.082 | 0.000 | 0.448 | 0.117 | 0.041 | 0.080 | 0.117 | 0.000 | 0.077 | 0.043 | 0.081 | 0.000 | 0.000 | 0.000 |
| RM241 | 0.382 | 0.407 | 0.619 | 0.320 | 0.083 | 0.227 | 0.219 | 0.402 | 0.156 | 0.100 | 0.082 | 0.000 | 0.286 | 0.623 | 0.260 | 0.080 | 0.170 | 0.153 | 0.621 | 0.595 | 0.518 | 0.153 | 0.197 | 0.000 |
| RM586 | 0.516 | 0.464 | 0.455 | 0.322 | 0.420 | 0.487 | 0.663 | 0.615 | 0.405 | 0.320 | 0.042 | 0.652 | 0.249 | 0.041 | 0.413 | 0.000 | 0.617 | 0.000 | 0.186 | 0.000 | 0.041 | 0.000 | 0.223 | 0.538 |
| RM336 | 0.306 | 0.483 | 0.343 | 0.124 | 0.297 | 0.084 | 0.489 | 0.498 | 0.000 | 0.156 | 0.500 | 0.041 | 0.174 | 0.520 | 0.041 | 0.385 | 0.081 | 0.207 | 0.078 | 0.213 | 0.080 | 0.041 | 0.121 | 0.000 |
| RM180 | 0.000 | 0.499 | 0.042 | 0.022 | 0.000 | 0.000 | 0.395 | 0.391 | 0.506 | 0.435 | 0.138 | 0.153 | 0.558 | 0.207 | 0.294 | 0.041 | 0.456 | 0.081 | 0.000 | 0.000 | 0.193 | 0.137 | 0.042 | 0.000 |
| RM223 | 0.233 | 0.044 | 0.000 | 0.000 | 0.000 | 0.000 | 0.041 | 0.044 | 0.000 | 0.510 | 0.384 | 0.080 | 0.369 | 0.041 | 0.284 | 0.305 | 0.489 | 0.254 | 0.215 | 0.340 | 0.344 | 0.000 | 0.283 | 0.187 |
| RM257 | 0.142 | 0.165 | 0.101 | 0.165 | 0.480 | 0.485 | 0.278 | 0.269 | 0.041 | 0.000 | 0.120 | 0.117 | 0.227 | 0.530 | 0.633 | 0.570 | 0.041 | 0.041 | 0.314 | 0.370 | 0.351 | 0.041 | 0.283 | 0.000 |
| RM333 | 0.748 | 0.578 | 0.082 | 0.000 | 0.567 | 0.592 | 0.413 | 0.494 | 0.627 | 0.551 | 0.436 | 0.000 | 0.576 | 0.041 | 0.835 | 0.707 | 0.254 | 0.312 | 0.215 | 0.237 | 0.440 | 0.223 | 0.000 | 0.041 |
| RM287 | 0.174 | 0.000 | 0.000 | 0.000 | 0.000 | 0.000 | 0.000 | 0.000 | 0.000 | 0.375 | 0.102 | 0.156 | 0.119 | 0.041 | 0.042 | 0.491 | 0.364 | 0.187 | 0.113 | 0.083 | 0.254 | 0.041 | 0.254 | 0.000 |
| RM247 | 0.174 | 0.000 | 0.042 | 0.000 | 0.423 | 0.499 | 0.041 | 0.000 | 0.000 | 0.353 | 0.263 | 0.041 | 0.081 | 0.080 | 0.353 | 0.536 | 0.595 | 0.317 | 0.186 | 0.198 | 0.291 | 0.153 | 0.235 | 0.249 |
| **总计Total** | **2.331** | **2.062** | **2.299** | **1.528** | **1.322** | **1.282** | **2.085** | **2.367** | **1.630** | **1.988** | **1.628** | **1.084** | **3.021** | **3.088** | **2.767** | **2.311** | **2.487** | **1.158** | **2.182** | **2.657** | **2.253** | **0.644** | **1.389** | **1.056** |
| **平均Mean** | **0.264** | **0.203** | **0.186** | **0.118** | **0.178** | **0.183** | **0.195** | **0.220** | **0.174** | **0.251** | **0.187** | **0.099** | **0.292** | **0.250** | **0.307** | **0.311** | **0.285** | **0.152** | **0.207** | **0.244** | **0.249** | **0.082** | **0.144** | **0.104** |

（continued）Tab.4 Nei’s genetic diversity (He) index for the rice landraces collected in different periods

| Locus | **Nei's genetic diversity index (He)** | | | | | | | | | | | | | | | | | | | | | | | |
| --- | --- | --- | --- | --- | --- | --- | --- | --- | --- | --- | --- | --- | --- | --- | --- | --- | --- | --- | --- | --- | --- | --- | --- | --- |
|  | Xiangnuogu | | Nuogu1 | | Nuogu2 | | Xiaowuzui | | Dibaigu | | Paozhugu | | Bendidanuo | | Honggu | | Heigu | | Xihongmi | | Danuo1 | | Danuo2 | |
|  | 2014 | 1980 | 2014 | 1980 | 2014 | 1980 | 2014 | 1980 | 2014 | 1980 | 2014 | 1980 | 2014 | 1980 | 2014 | 1980 | 2014 | 1980 | 2014 | 1980 | 2014 | 1980 | 2014 | 1980 |
| RM449 | 0.117 | 0.000 | 0.081 | 0.100 | 0.000 | 0.080 | 0.451 | 0.041 | 0.000 | 0.041 | 0.523 | 0.278 | 0.042 | 0.000 | 0.081 | 0.278 | 0.041 | 0.392 | 0.000 | 0.000 | 0.000 | 0.000 | 0.000 | 0.000 |
| RM48 | 0.041 | 0.081 | 0.081 | 0.190 | 0.080 | 0.000 | 0.377 | 0.190 | 0.353 | 0.000 | 0.646 | 0.000 | 0.082 | 0.000 | 0.041 | 0.364 | 0.000 | 0.080 | 0.000 | 0.000 | 0.226 | 0.187 | 0.375 | 0.000 |
| RM251 | 0.000 | 0.000 | 0.157 | 0.209 | 0.000 | 0.061 | 0.041 | 0.041 | 0.000 | 0.000 | 0.800 | 0.335 | 0.160 | 0.000 | 0.000 | 0.000 | 0.041 | 0.496 | 0.119 | 0.000 | 0.000 | 0.219 | 0.444 | 0.000 |
| RM471 | 0.000 | 0.000 | 0.041 | 0.041 | 0.000 | 0.000 | 0.000 | 0.041 | 0.153 | 0.000 | 0.542 | 0.350 | 0.042 | 0.000 | 0.000 | 0.000 | 0.000 | 0.190 | 0.000 | 0.000 | 0.353 | 0.153 | 0.000 | 0.041 |
| RM241 | 0.603 | 0.153 | 0.100 | 0.155 | 0.000 | 0.021 | 0.633 | 0.190 | 0.585 | 0.531 | 0.832 | 0.190 | 0.082 | 0.080 | 0.499 | 0.678 | 0.117 | 0.411 | 0.000 | 0.000 | 0.000 | 0.000 | 0.573 | 0.156 |
| RM586 | 0.312 | 0.353 | 0.041 | 0.080 | 0.000 | 0.061 | 0.219 | 0.000 | 0.061 | 0.061 | 0.812 | 0.155 | 0.042 | 0.000 | 0.041 | 0.354 | 0.120 | 0.423 | 0.061 | 0.413 | 0.153 | 0.187 | 0.358 | 0.000 |
| RM336 | 0.119 | 0.000 | 0.021 | 0.155 | 0.000 | 0.000 | 0.000 | 0.000 | 0.119 | 0.000 | 0.780 | 0.330 | 0.042 | 0.080 | 0.545 | 0.249 | 0.249 | 0.292 | 0.170 | 0.021 | 0.351 | 0.506 | 0.430 | 0.515 |
| RM180 | 0.633 | 0.576 | 0.041 | 0.156 | 0.000 | 0.000 | 0.495 | 0.000 | 0.203 | 0.170 | 0.125 | 0.234 | 0.062 | 0.219 | 0.080 | 0.249 | 0.000 | 0.330 | 0.497 | 0.187 | 0.249 | 0.000 | 0.153 | 0.000 |
| RM223 | 0.080 | 0.187 | 0.225 | 0.281 | 0.506 | 0.497 | 0.385 | 0.000 | 0.154 | 0.000 | 0.670 | 0.041 | 0.654 | 0.190 | 0.430 | 0.305 | 0.420 | 0.401 | 0.000 | 0.249 | 0.041 | 0.330 | 0.474 | 0.000 |
| RM257 | 0.041 | 0.499 | 0.080 | 0.155 | 0.041 | 0.219 | 0.480 | 0.489 | 0.742 | 0.231 | 0.493 | 0.497 | 0.178 | 0.000 | 0.041 | 0.398 | 0.041 | 0.430 | 0.000 | 0.080 | 0.225 | 0.080 | 0.278 | 0.080 |
| RM333 | 0.423 | 0.234 | 0.119 | 0.194 | 0.041 | 0.080 | 0.646 | 0.000 | 0.458 | 0.533 | 0.479 | 0.465 | 0.141 | 0.346 | 0.187 | 0.041 | 0.000 | 0.000 | 0.081 | 0.534 | 0.000 | 0.431 | 0.117 | 0.000 |
| RM287 | 0.000 | 0.000 | 0.347 | 0.190 | 0.000 | 0.041 | 0.155 | 0.000 | 0.000 | 0.000 | 0.822 | 0.061 | 0.491 | 0.000 | 0.081 | 0.223 | 0.229 | 0.210 | 0.081 | 0.381 | 0.000 | 0.000 | 0.000 | 0.000 |
| RM247 | 0.041 | 0.296 | 0.041 | 0.041 | 0.000 | 0.061 | 0.681 | 0.661 | 0.228 | 0.081 | 0.742 | 0.187 | 0.343 | 0.000 | 0.000 | 0.000 | 0.080 | 0.081 | 0.081 | 0.041 | 0.170 | 0.223 | 0.000 | 0.000 |
| **总计Total** | **1.945** | **1.848** | **0.866** | **1.523** | **0.627** | **0.937** | **3.080** | **0.992** | **2.371** | **1.034** | **6.223** | **2.410** | **1.385** | **0.569** | **1.756** | **2.875** | **1.029** | **3.444** | **0.846** | **0.949** | **1.597** | **1.661** | **3.084** | **0.792** |
| **平均Mean** | **0.185** | **0.183** | **0.106** | **0.150** | **0.051** | **0.086** | **0.351** | **0.127** | **0.235** | **0.127** | **0.636** | **0.240** | **0.182** | **0.070** | **0.156** | **0.242** | **0.103** | **0.287** | **0.084** | **0.147** | **0.136** | **0.178** | **0.246** | **0.061** |

Tab.5 Shannon’s genetic diversity index (I) for the rice landraces collected in different periods

| Locus | **Shannon's Information index（I）** | | | | | | | | | | | | | | | | | | | | | | | |
| --- | --- | --- | --- | --- | --- | --- | --- | --- | --- | --- | --- | --- | --- | --- | --- | --- | --- | --- | --- | --- | --- | --- | --- | --- |
|  | Baixianghe | | Ronghe | | Danuo | | Heinuo | | Dalaogeng | | Huangnuogu | | Laogengbaijiao | | Yaduogu | | Laogenghongjiao | | Huagu | | Lengshuigu | | Jiuyuenuo | |
|  | 2014 | 1980 | 2014 | 1980 | 2014 | 1980 | 2014 | 1980 | 2014 | 1980 | 2014 | 1980 | 2014 | 1980 | 2014 | 1980 | 2014 | 1980 | 2014 | 1980 | 2014 | 1980 | 2014 | 1980 |
| RM449 | 0.317 | 0.000 | 0.000 | 0.000 | 0.105 | 0.000 | 0.000 | 0.215 | 0.058 | 0.000 | 0.456 | 0.101 | 0.159 | 0.000 | 0.000 | 0.000 | 0.000 | 0.000 | 0.324 | 0.241 | 0.101 | 0.000 | 0.000 | 0.000 |
| RM48 | 0.365 | 0.000 | 0.176 | 0.000 | 0.000 | 0.000 | 0.000 | 0.107 | 0.101 | 0.000 | 0.000 | 0.000 | 0.415 | 0.858 | 0.377 | 1.233 | 0.475 | 0.234 | 0.293 | 0.446 | 0.584 | 0.287 | 0.220 | 0.101 |
| RM251 | 0.325 | 0.000 | 0.679 | 0.580 | 0.000 | 0.000 | 0.000 | 0.000 | 0.101 | 0.512 | 0.000 | 0.000 | 0.689 | 0.837 | 1.070 | 0.513 | 0.584 | 0.600 | 0.993 | 1.481 | 0.435 | 0.274 | 0.339 | 0.561 |
| RM471 | 0.510 | 0.000 | 0.316 | 0.378 | 0.000 | 0.000 | 0.000 | 0.000 | 0.752 | 0.305 | 0.176 | 0.000 | 0.778 | 0.234 | 0.101 | 0.173 | 0.234 | 0.000 | 0.168 | 0.105 | 0.202 | 0.000 | 0.000 | 0.000 |
| RM241 | 0.740 | 0.597 | 1.025 | 0.500 | 0.179 | 0.387 | 0.377 | 0.656 | 0.345 | 0.231 | 0.176 | 0.000 | 0.531 | 1.090 | 0.515 | 0.173 | 0.311 | 0.287 | 1.242 | 1.057 | 0.778 | 0.287 | 0.451 | 0.000 |
| RM586 | 0.867 | 0.725 | 0.862 | 0.627 | 0.755 | 0.839 | 1.232 | 1.119 | 0.732 | 0.602 | 0.103 | 1.076 | 0.415 | 0.101 | 0.604 | 0.000 | 1.024 | 0.000 | 0.431 | 0.000 | 0.101 | 0.000 | 0.382 | 0.837 |
| RM336 | 0.671 | 0.924 | 0.641 | 0.245 | 0.567 | 0.203 | 0.748 | 0.691 | 0.000 | 0.345 | 0.693 | 0.101 | 0.371 | 1.037 | 0.101 | 0.574 | 0.202 | 0.410 | 0.196 | 0.403 | 0.173 | 0.101 | 0.278 | 0.000 |
| RM180 | 0.000 | 0.692 | 0.103 | 0.061 | 0.000 | 0.000 | 0.584 | 0.580 | 0.855 | 0.755 | 0.265 | 0.287 | 0.897 | 0.410 | 0.613 | 0.116 | 0.806 | 0.197 | 0.000 | 0.000 | 0.433 | 0.305 | 0.103 | 0.000 |
| RM223 | 0.395 | 0.109 | 0.000 | 0.000 | 0.000 | 0.000 | 0.101 | 0.107 | 0.000 | 0.769 | 0.698 | 0.173 | 0.647 | 0.101 | 0.513 | 0.483 | 0.893 | 0.475 | 0.421 | 0.524 | 0.746 | 0.000 | 0.456 | 0.334 |
| RM257 | 0.271 | 0.305 | 0.208 | 0.350 | 0.673 | 0.678 | 0.451 | 0.496 | 0.101 | 0.000 | 0.237 | 0.234 | 0.485 | 0.875 | 1.050 | 0.947 | 0.101 | 0.101 | 0.645 | 0.764 | 0.708 | 0.101 | 0.456 | 0.000 |
| RM333 | 1.542 | 0.968 | 0.206 | 0.000 | 1.180 | 1.162 | 0.604 | 0.687 | 1.194 | 1.021 | 0.774 | 0.000 | 1.008 | 0.101 | 1.977 | 1.394 | 0.475 | 0.548 | 0.421 | 0.519 | 0.858 | 0.433 | 0.000 | 0.101 |
| RM287 | 0.317 | 0.000 | 0.000 | 0.000 | 0.000 | 0.000 | 0.000 | 0.000 | 0.000 | 0.562 | 0.244 | 0.345 | 0.274 | 0.101 | 0.103 | 0.724 | 0.551 | 0.334 | 0.227 | 0.179 | 0.475 | 0.101 | 0.421 | 0.000 |
| RM247 | 0.317 | 0.000 | 0.118 | 0.000 | 0.615 | 0.692 | 0.101 | 0.000 | 0.000 | 0.538 | 0.510 | 0.101 | 0.202 | 0.173 | 0.538 | 0.834 | 1.125 | 0.582 | 0.420 | 0.398 | 0.565 | 0.287 | 0.552 | 0.415 |
| **总计Total** | **4.460** | **3.351** | **4.009** | **2.741** | **2.278** | **2.107** | **3.492** | **3.970** | **3.045** | **3.518** | **2.804** | **1.972** | **5.387** | **5.543** | **4.944** | **4.212** | **4.630** | **2.304** | **4.711** | **5.020** | **4.262** | **1.355** | **2.686** | **1.833** |
| **平均Mean** | **0.510** | **0.332** | **0.333** | **0.211** | **0.313** | **0.305** | **0.323** | **0.358** | **0.326** | **0.434** | **0.333** | **0.186** | **0.529** | **0.455** | **0.582** | **0.551** | **0.522** | **0.290** | **0.445** | **0.471** | **0.474** | **0.167** | **0.281** | **0.181** |

（continued）Tabl.5 Shannon’s genetic diversity index (I) for the rice landraces collected in different periods

| 引物 Locus | **Shannon's Information index （I）** | | | | | | | | | | | | | | | | | | | | | | | |
| --- | --- | --- | --- | --- | --- | --- | --- | --- | --- | --- | --- | --- | --- | --- | --- | --- | --- | --- | --- | --- | --- | --- | --- | --- |
|  | Xiangnuogu | | Nuogu1 | | Nuogu2 | | Xiaowuzui | | Dibaigu | | Paozhugu | | Bendidanuo | | Honggu | | Heigu | | Xihongmi | | Danuo1 | | Danuo2 | |
|  | 2014 | 1980 | 2014 | 1980 | 2014 | 1980 | 2014 | 1980 | 2014 | 1980 | 2014 | 1980 | 2014 | 1980 | 2014 | 1980 | 2014 | 1980 | 2014 | 1980 | 2014 | 1980 | 2014 | 1980 |
| RM449 | 0.234 | 0.000 | 0.202 | 0.231 | 0.000 | 0.173 | 0.644 | 0.101 | 0.000 | 0.101 | 0.976 | 0.451 | 0.103 | 0.000 | 0.202 | 0.451 | 0.101 | 0.675 | 0.000 | 0.000 | 0.000 | 0.000 | 0.000 | 0.000 |
| RM48 | 0.101 | 0.202 | 0.202 | 0.386 | 0.173 | 0.000 | 0.643 | 0.386 | 0.538 | 0.000 | 1.253 | 0.000 | 0.206 | 0.000 | 0.101 | 0.671 | 0.000 | 0.173 | 0.000 | 0.000 | 0.456 | 0.334 | 0.562 | 0.000 |
| RM251 | 0.000 | 0.000 | 0.362 | 0.443 | 0.000 | 0.139 | 0.101 | 0.101 | 0.000 | 0.000 | 1.961 | 0.518 | 0.380 | 0.000 | 0.000 | 0.000 | 0.101 | 1.016 | 0.274 | 0.000 | 0.000 | 0.377 | 0.637 | 0.000 |
| RM471 | 0.000 | 0.000 | 0.101 | 0.101 | 0.000 | 0.000 | 0.000 | 0.101 | 0.287 | 0.000 | 1.159 | 0.594 | 0.103 | 0.000 | 0.000 | 0.000 | 0.000 | 0.386 | 0.000 | 0.000 | 0.538 | 0.287 | 0.000 | 0.101 |
| RM241 | 1.101 | 0.287 | 0.240 | 0.334 | 0.000 | 0.058 | 1.198 | 0.386 | 1.029 | 0.829 | 1.913 | 0.386 | 0.206 | 0.173 | 0.692 | 1.280 | 0.234 | 0.802 | 0.000 | 0.000 | 0.000 | 0.000 | 1.054 | 0.342 |
| RM586 | 0.548 | 0.538 | 0.101 | 0.173 | 0.000 | 0.139 | 0.377 | 0.000 | 0.159 | 0.159 | 1.824 | 0.334 | 0.103 | 0.000 | 0.101 | 0.635 | 0.303 | 0.770 | 0.139 | 0.604 | 0.287 | 0.334 | 0.581 | 0.000 |
| RM336 | 0.274 | 0.000 | 0.058 | 0.334 | 0.000 | 0.000 | 0.000 | 0.000 | 0.274 | 0.000 | 1.673 | 0.512 | 0.118 | 0.173 | 0.909 | 0.415 | 0.415 | 0.566 | 0.311 | 0.058 | 0.652 | 0.930 | 0.621 | 0.880 |
| RM180 | 1.043 | 1.056 | 0.101 | 0.345 | 0.000 | 0.000 | 0.688 | 0.000 | 0.356 | 0.311 | 0.328 | 0.397 | 0.162 | 0.377 | 0.173 | 0.415 | 0.000 | 0.512 | 0.690 | 0.334 | 0.415 | 0.000 | 0.287 | 0.000 |
| RM223 | 0.173 | 0.334 | 0.467 | 0.640 | 0.766 | 0.690 | 0.634 | 0.000 | 0.318 | 0.000 | 1.142 | 0.101 | 1.078 | 0.386 | 0.703 | 0.483 | 0.712 | 0.713 | 0.000 | 0.415 | 0.101 | 0.512 | 0.667 | 0.000 |
| RM257 | 0.101 | 0.865 | 0.173 | 0.334 | 0.101 | 0.377 | 0.837 | 0.762 | 1.620 | 0.572 | 0.942 | 0.690 | 0.397 | 0.000 | 0.101 | 0.703 | 0.101 | 0.765 | 0.000 | 0.173 | 0.447 | 0.173 | 0.451 | 0.173 |
| RM333 | 0.770 | 0.397 | 0.274 | 0.444 | 0.101 | 0.173 | 1.283 | 0.000 | 0.864 | 0.945 | 0.957 | 0.813 | 0.336 | 0.777 | 0.334 | 0.101 | 0.000 | 0.000 | 0.202 | 0.880 | 0.000 | 0.771 | 0.234 | 0.000 |
| RM287 | 0.000 | 0.000 | 0.664 | 0.386 | 0.000 | 0.101 | 0.334 | 0.000 | 0.000 | 0.000 | 1.873 | 0.159 | 0.871 | 0.000 | 0.202 | 0.433 | 0.531 | 0.461 | 0.202 | 0.661 | 0.000 | 0.000 | 0.000 | 0.000 |
| RM247 | 0.101 | 0.631 | 0.101 | 0.101 | 0.000 | 0.139 | 1.242 | 1.126 | 0.503 | 0.202 | 1.542 | 0.334 | 0.587 | 0.000 | 0.000 | 0.000 | 0.173 | 0.202 | 0.202 | 0.101 | 0.311 | 0.433 | 0.000 | 0.000 |
| **总计Total** | **3.575** | **3.282** | **2.008** | **3.320** | **1.040** | **1.576** | **5.122** | **1.839** | **4.581** | **1.973** | **13.170** | **3.982** | **2.854** | **1.110** | **2.983** | **5.053** | **1.968** | **6.378** | **1.414** | **1.584** | **2.897** | **2.947** | **4.859** | **1.496** |
| **平均Mean** | **0.342** | **0.332** | **0.234** | **0.327** | **0.088** | **0.153** | **0.614** | **0.228** | **0.458** | **0.240** | **1.349** | **0.407** | **0.358** | **0.145** | **0.271** | **0.430** | **0.206** | **0.542** | **0.155** | **0.248** | **0.247** | **0.319** | **0.392** | **0.115** |
